# Identification of differentially expressed genes in human breast cancer cells induced by 4-hydroxyltamoxifen and elucidation of their pathophysiological relevance and mechanisms

**DOI:** 10.18632/oncotarget.23504

**Published:** 2017-12-20

**Authors:** Qi Fang, Shuang Yao, Guanghua Luo, Xiaoying Zhang

**Affiliations:** ^1^ Department of Breast Surgery, The Third Affiliated Hospital of Soochow University, Changzhou 213003, P.R. China; ^2^ Comprehensive Laboratory, The Third Affiliated Hospital of Soochow University, Changzhou 213003, P.R. China

**Keywords:** breast cancer, MCF-7, 4-hydroxyl tamoxifen, STAT1, STAT2

## Abstract

While tamoxifen (TAM) is used for treating estrogen receptor (ER)a-positive breast cancer patients, its anti-breast cancer mechanisms are not completely elucidated. This study aimed to examine effects of 4-hydroxyltamoxifen (4-OH-TAM) on ER-positive (ER^+^) breast cancer MCF-7 cell growth and gene expression profiles. MCF-7 cell growth was inhibited by 4-OH-TAM dose-dependently with IC_50_ of 29 μM. 332 genes were up-regulated while 320 genes were down-regulated. The mRNA levels of up-regulated genes including STAT1, STAT2, EIF2AK2, TGM2, DDX58, PARP9, SASH1, RBL2 and USP18 as well as down-regulated genes including CCDN1, S100A9, S100A8, ANXA1 and PGR were confirmed by quantitative real-time PCR (qRT-PCR). In human breast tumor tissues, mRNA levels of EIF2Ak2, USP18, DDX58, RBL2, STAT2, PGR, S1000A9, and CCND1 were significantly higher in ER^+^- than in ER^-^-breast cancer tissues. The mRNA levels of EIF2AK2, TGM2, USP18, DDX58, PARP9, STAT2, STAT1, PGR and CCND1 were all significantly higher in ER^+^-tumor tissues than in their corresponding tumor-adjacent tissues. These genes, except PGR and CCND1 which were down-regulated, were also up-regulated in ER^+^ MCF-7 cells by 4-OH-TAM. Total 14 genes mentioned above are involved in regulation of cell proliferation, apoptosis, cell cycles, and estrogen and interferon signal pathways. Bioinformatics analysis also revealed other novel and important regulatory factors that are associated with these genes and involved in the mentioned functional processes. This study has paved a foundation for elucidating TAM anti-breast cancer mechanisms in E2/ER-dependent and independent pathways.

## INTRODUCTION

Breast cancer is the second most common cause of cancer-related death among women in the world [1, 2]. Approximately 246,660 new cases of invasive breast cancer, including 61,000 cases of carcinoma *in situ* in U.S. women were estimated, among which, 40,450 patients would die in 2016 [3]. Approximately 1.7 million new cases of breast cancer occurred among women worldwide in 2012 [4]. Breast cancer is also the most commonly diagnosed cancer in women in mainland China with the incident rate of 268.6/100,000 population, which has been increased by 3.9% annually [5].

Breast cancer exhibits remarkable clinical and molecular heterogeneity. Based on gene expression profiles and the status of hormone receptors, e.g. estrogen receptors alpha and beta (ERα and ERβ), progesterone receptor (PR) and overexpression of human epidermal growth factor receptor 2 (HER2), breast cancer is classified into five subtypes: i.e. luminal A(ER^+^ and/or PR^+^, HER2^-^, Ki-67<14), luminal B (ER^+^ and/or PR^+^, HER2^-^, Ki-67≥14; ER^+^ and/or PR^+^, HER2^+^), HER2 overexpression (ER^-^/PR^-^/HER2^+^), triple negative breast cancer (ER^-^/PR^-^/HER2^-^) (TNBC) and normal breast-like breast cancer [6]. Luminal A and TNBC account for about 60-70% and 15-20% of total breast cancer cases, respectively [6, 7]. Recent studies [8, 9] have identified long-non-coding RNAs as the prognostic markers for prediction of the risk of tumor recurrence of breast cancer patients. Low oncogenic GTP activity, low ubiquitin/proteasome degradation, effective protection from oxidative damage and tightly immune response have been identified as the prognostic markers for TNBC [10]. While clinical differences among these subtypes have been well studied, their etiologic heterogeneity has not been fully addressed. Several factors associated with increased levels, prolonged exposure to estrogen and the status of ERα and ERβ are significantly associated with risk of ER-positive breast cancer [11–13].

17β-estradiol (E2) plays important roles in regulating cell proliferation, differentiation, and development at puberty and during sexual maturity. These effects are mediated via ERα and ERβ[14] as well as other ER-related factors/receptors, including ER-related receptor [15] and G-protein coupled receptors [16]. However, prolonged exposure to excess amount of E2 has been regarded as a key factor associated with the increased risk of breast cancer [17]. The pro-carcinogenetic effects of E2 are generally attributed to (a) E2/ER-mediated cell proliferation [17, 18]; (b) gene mutation initiated by catechol metabolites via cytochrome P450-mediated activation of E2 metabolism [17]; (c) aneuploidy through activation of aurora A [19] and (d) changes in chromosomal structures induced by E2 via ERR in both ER^+^ - and ER^-^- breast cancer cells [20]. ERα plays an important role in estrogen carcinogenesis of breast cancer [21]. Therefore, reduction of estrogen levels by inhibiting estrogen biosynthesis with aromatase inhibitor and/or blockage of E2/ERα-mediated signaling pathways with selective ER modulators or selective ER down-regulator have become an integral part of the management of hormone-dependent and ERα-positive breast cancer [21, 22].

Endocrine therapies are one of the effective and systemic treatments for patients with ERα-positive breast cancer. To date, tamoxifen (TAM), an E2 antagonist with high affinity to ERα present in 60-70% of breast cancer patients, is the most commonly used medicine of patients with ERα-positive breast cancer. Several clinical trials [23–30] indicated: (a) treatment of invasive breast cancer patients with TAM significantly reduced the recurrence and death rate by 26% and 14%, after a median follow-up of 10 years; (b) contralateral cancer risk, a metastatic spread of first breast cancer, was reduced by 50% after 5-year TAM treatment; (c) an reduction of overall breast cancer incidence by 38% within the first 10 years after TAM treatment for > 5 years. An extended 16-year follow-up of IBIS breast cancer prevention trial also revealed a substantial reduction in risk in women with invasive ER-positive breast cancer and ductal carcinomas *in situ,* which was not seen in patients with ER^-^-breast cancer. Five years of adjuvant TAM safely reduced 15-year risks of breast cancer recurrence and death. ER status was the only recorded factor predictive of the proportional reductions [31]. Together, these lines of evidence have demonstrated that TAM can offer a long-term protection after treatment cessation and thus, can improve prevention for this subtype of breast cancer.

The anti-breast cancer effects of TAM are mainly attributed to its anti-estrogenic activity via its competitive inhibition on E2/ERα signal pathways [32]. TAM interferes with the expression of E2-regulated genes. Its effects on gene expression in breast cancer cells have been analyzed and a number of TAM-modulated genes [33–35] and micro RNAs [36] have been identified. It is likely that beside its inhibitory effects on E2/ERα-dependent gene expression profiles, TAM can also cause inhibitory and activating effects on other important pathways in breast cancer cells via E2/ERα-independent manner. Thus, the present study aimed to further examine the effects of TAM on ER positive (ER^+^) MCF-7 cell growth, to identify the novel gene expression profiles in MCF-7 cells caused by TAM treatment. Furthermore, we also attempted to examine the transcriptional profiles of TAM-regulated genes in both ER^+^- and ER^-^-breast cancer cell lines and to compare their transcriptional expression profiles in ER^+^- and ER^-^-breast tumor tissues with those of their corresponding tumor-adjacent tissues obtained from patients with breast cancer, aiming to reveal the pathophysiological relevance of TAM-regulated genes and mechanisms underlying TAM-anti-breast cancer effects.

## RESULTS

### Inhibitory effects of 4-OH-TAM on growth and proliferation of MCF-7 cells

**Figure 1:**
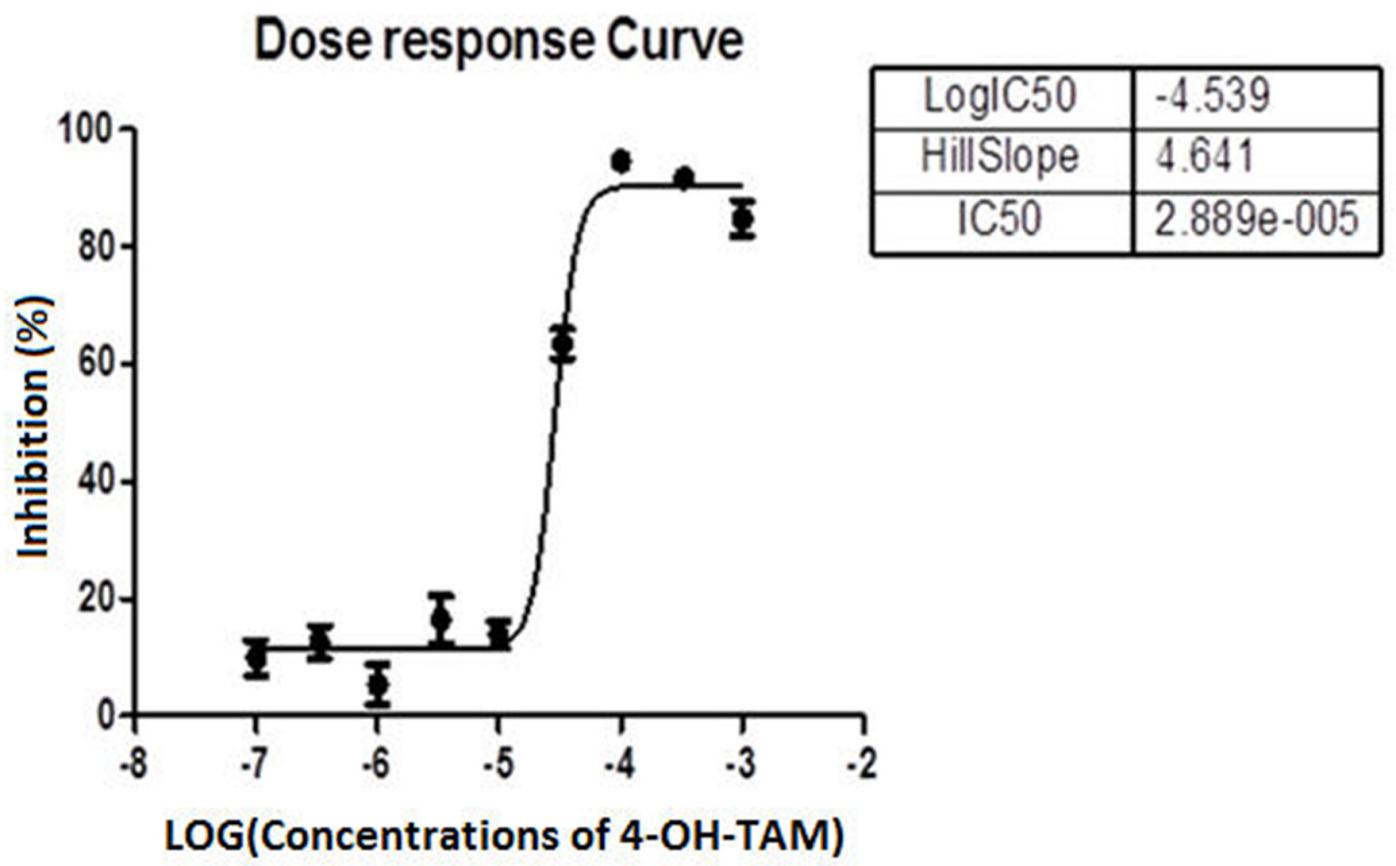
Dose response curve showing the percentage of inhibition of MCF-7 cell growth versus log concentrations of 4-OH-TAM

After MCF-7 cells were treated with 4-OH-TAM at 0 to 3.33 × 10^-3^ M (Table 1) for 72h, the inhibitory effects of 4-OH-TAM on MCF-7 cells were determined with CCK-8 kit. The results shown in Table 2 indicated that 4-OH-TAM caused a potent inhibition on MCF-7 cell growth in a dose-dependent manner. At 1 × 10^-7^ M, the MCF-7 cell growth rate was reduced by 9.9%. When the concentration of 4–OH-TAM was increased to 3.33 × 10^-4^ M, the inhibition rate reached 94.4%, the maximal value. Figure 1 showed the dose-dependent curve of MCF-7 cell inhibition in relative to the log concentrations of 4-OH-TAM, which displayed “S” shape. The IC_50_ was determined to be 29μM.

**Table 1:** Treatment of MCF-7 cells with 4-OH-TAM at the indicated concentrations

Group	Cell number/well		Medium	TAM concentration (M)
	A	B		
1	4000 cells	8000 cells	Complete DMEM	0
2	4000 cells	8000 cells	Complete DMEM	1×10^-7^
3	4000 cells	8000 cells	Complete DMEM	3.33 × 10^-7^
4	4000 cells	8000 cells	Complete DMEM	1×10^-6^
5	4000 cells	8000 cells	Complete DMEM	3.33 × 10^-6^
6	4000 cells	8000 cells	Complete DMEM	1×10^-5^
7	4000 cells	8000 cells	Complete DMEM	3.33 × 10^-5^
8	4000 cells	8000 cells	Complete DMEM	1×10^-4^
9	4000 cells	8000 cells	Complete DMEM	3.33 × 10^-4^
10	4000 cells	8000 cells	Complete DMEM	1×10^-3^
11	4000 cells	8000 cells	Complete DMEM	3.33×10^-3^

MCF-7 cell suspension containing 200 mL of 4000 cells/well and 8000 cells/well corresponding to 20% and 40% confluences per wells of the 96-well plate were seeded. The cells in each group were treated with 4-OH-TAM at the indicated concentrations for 72 h.

**Table 2:** Dose-dependent inhibitory effects of 4-OH-TAM on MCF-7 cell growth

Group	Treatment	% of inhibition
1	Control (NC)	0
2	1×10^-7^	9.9
3	3.33 × 10^-7^	12.5
4	1×10^-6^	5.4
5	3.33 × 10^-6^	16.4
6	1×10^-5^	13.8
7	3.33 × 10^-5^	63.4
8	1×10^-4^	63.4
9	3.33 × 10^-4^	94.4
10	1×10^-3^	91.7
11	3.33×10^-3^	85.5

The cells in each group were treated with 4-OH-TAM at the indicated concentrations for 72 h. The cell viability in each group was determined by CCK8 assay with commercial CCK8 kit.

### Microarray analysis for differentially expressed genes between 4-OH-TAM-treated group and NC groups

**Figure 2:**
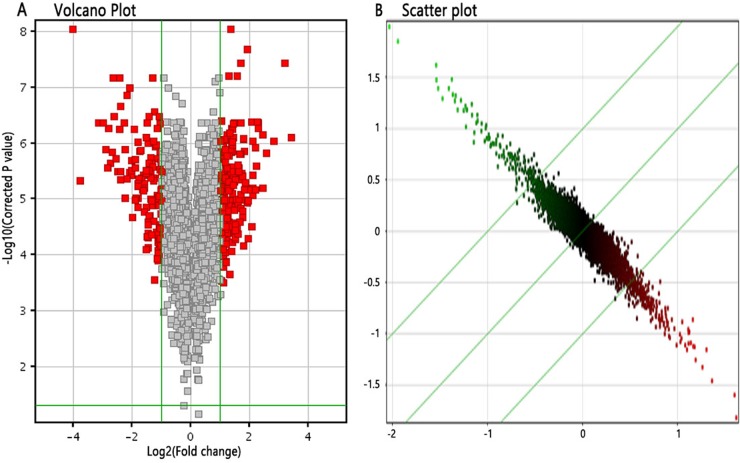
Differentially expressed genes between 4-OH-TAM-treated group and NC groups **(A)** Volcano Plot, which demonstrated the distribution of the differentially expressed genes between TAM-treated group and control group. The X-axis represents the logarithm conversion of the fold difference to base 2 and the Y-axis represents the logarithm conversion of the corrected significant levels to base 10. The red color represents all the probes with fold difference >1.5 at significant level of P<0.05. **(B)** Scatter plot, which exhibited the distribution of the signals between TAM-treated group and control group in Cartesian coordinate plane. The X-axis represents TAM-treated group, and the Y-axis represents the control group. The ordinate value and the abscissa of each spot represent the expression values of one probe in TAM-treated group and control group. The parts above the green lines represent the down-regulated probes in relative to the control group. The parts underneath the green lines represent the up-regulated probes as compared to those of the control group.

We applied GeneChip^®^ PrimeView^™^ Human Gene Expression Array to investigate the gene expression profiles of MCF-7 cells induced by 4-OH-TAM at 1×10^-7^ M for 72 h. After validating the quality of microarray data (Supplementary Figure 1), we draw Volcano plot (Figure 2A), Scatter plot (Figure 2B). Volcano plot (Figure 2A) demonstrated the distribution of the differentially expressed genes between 4-OH-TAM-treated group and NC group. The red color represents all the genes whose expression levels were of >1.5 fold-difference at significant level of *P*<0.05. Scatter plot (Figure 2B) exhibited the distribution of the signals between 4-OH-TAM-treated group and control group in Cartesian coordinate plane. The parts above the upper green lines represent the down-regulated probes in relative to those of the control group while the parts below the lower green lines represent the up-regulated probes in relative to those of the control group.

The screening criteria for differentially expressed genes were set as follows: there was significant > 1.5 folds difference in gene expression level between two groups with *P*<0.05. Based on these criteria, the differentially expressed genes were identified. As compared to those of NC group, a total of 332 up-regulated genes and a total of 320 down-regulated genes in 4-OH-TAM-treated group were identified. The up-regulated genes included STAT1, STAT2, EIF2AK2, TGM2, DDX58, PARP9, SASH1, RBL2 and USP18 and their expression levels were up-regulated by 1.581 to 2.337 folds by 4-OH-TAM. The down-regulated genes included CCDN1, S100A9, S100A8, ANXA1, and PGR and their mRNA levels were down-regulated by 1.709 to 4.753 folds by 4-OH-TAM (Table 3).

**Table 3:** Differentially expressed genes between 4-OH-TAM-treated group and NC Groups

Gene name	Fold change	Molecule type	FDR
STAT1	2.247	transcription regulator	3.216E-06
STAT2	1.581	transcription regulator	3.216E-06
EIF2AK2	1.765	kinase	2.767E-05
TGM2	1.923	enzyme	0.000141959
DDX58	2.337	enzyme	1.663E-06
PARP9	1.719	enzyme	4.047E-06
SASH1	1.990	other	1.255 E-05
RBL2	1.922	other	0.00026717
USP18	1.861	peptidase	5.454 E-06
CCND1	-1.709	transcription regulator	1.078E-05
S100A9	-3.560	other	9.507 E-07
S100A8	-4.562	other	1.370 E-07
ANXA1	-2.307	enzyme	3.927 E-05
PGR	-4.753	ligand-dependent nuclear receptor	1.270 E-06

### Verification of up-regulated and down-regulated genes using quantitative real-time PCR

**Figure 3:**
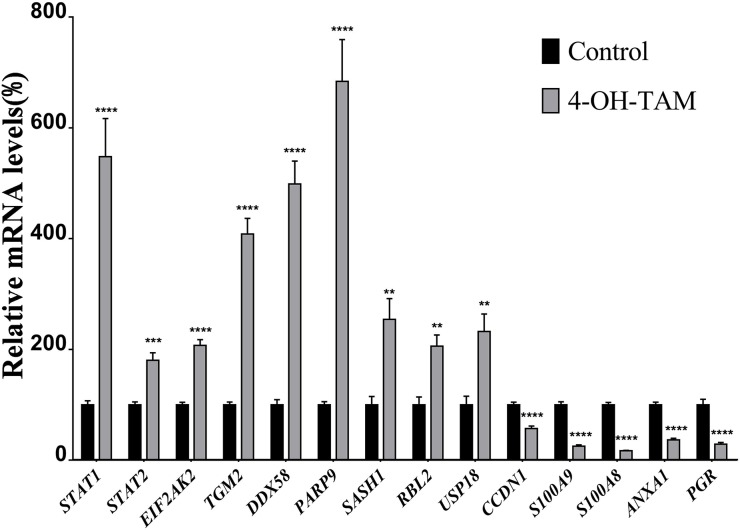
Relative mRNA levels of up-regulated and down-regulated genes induced by 4-OH-TAM treatment in MCF-7 cells MCF-7 cells were treated with or without 4-OH-TAM at 1.0×10^-7^M for 72 h before cells collection. Total RNA samples were isolated from 4-OH-TAM treated and NC groups and qRT-PCR analyses were performed as described in Materials and Method section. Data are presented as means ± SD. ^*^P <0.05, ^**^P <0.01, ^***^P <0.001 ^****^P <0.0001 vs. controls (n=7).

We applied quantitative real-time PCR (qRT-PCR) to further verify 14 representative up-regulated and down-regulated genes identified via microarray. The mRNA levels of the up-regulated genes, and those of the down-regulated genes were detected by qRT-PCR and the results shown in Figure 3 indicated that the mRNA levels of STAT1, STAT2, EIF2AK2, TGM2, DDX58, PARP9, SASH1, RBL2 and USP18 were significantly increased to 5.482, 1.806, 2.074, 4.087, 4.986, 6.840, 2.545, 2.057, and 3.806 folds, respectively, whereas those of CCDN1, S100A9, S100A8, ANXA1 and PGR were significantly reduced to 1.748, 3.924, 5.886, 2.723, and 3.443 folds, respectively, as compared to those of NC group. Among them, the mRNA levels of STAT1 TGM2, DDX58, PARP9 and USP18 were highly induced by 4-OH-TAM treatment while those of PRG, S100A8 and S100A9 were potently suppressed by 4-OH-TAM treatment. These results were totally consistent with those obtained with microarray, clearly confirming the reliability of gene expression profiles revealed by microarray analysis.

### The mRNA expression profiles of differentially expressed genes in both ER^+^- and ER^-^-breast cancer cell lines

**Figure 4:**
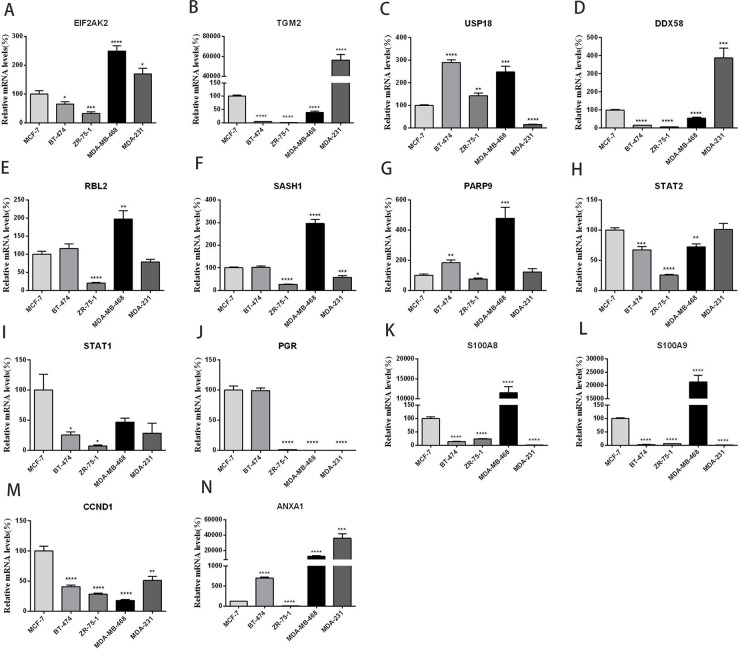
Relative mRNA levels of 14 genes **(panels A-N)** in three ER^+^ - and two ER^-^-breast cancer cell lines. MCF-7 and ZR-75-1: Luminal A subtype; BT-474: Luminal B subtype; MDA-MB-231 and MDA-MB-468: Basal-like subtype. Data are presented as means ± SEM (n=6). ^*^P <0.05, ^**^P <0.01, ^***^P <0.001 ^****^P <0.0001 vs. controls (MCF-7), was taken as100%.

To further verify the differentially expressed genes induced by 4-OH-TAM in MCF-7 cells and to understand their pathophysiological relevance in ER^+^- and ER^-^-breast cancer cells, we examined their mRNA levels in three ER^+^-breast cancer cell lines (MCF-7, BT-474 and ZR-75-1) and two ER^-^-breast cancer cell lines (MDA-MB-468 and MDA-MB-231) via qRT-PCR. MCF-7 is classified to Luminal A subtype; ZR-75-1 and BT-474 belong to Luminal B subtype; Both MDA-MB-231 and MDA-MB-468 are classified into basal-like subtype. Figure 4 showed the expression profiles of EIF2AK2 (A), TGM2 (B), USP-18 (C), DDX58 (D), RBL2 (E), SASH1(F), PARP9 (G), STAT2 (H), STAT1 (I), PGR (J), S100A8 (K), S100A9 (L), CCND1 (M) and ANXA1 (N) in these five breast cancer cell lines. Among three ER^+^-breast cancer cell lines, compared with those of MCF-7 cells, the mRNA levels of EIF2AK2 (*P*<0.05), TGM2 (*P*<0.0001), DDX58 (*P*<0.0001), STAT2 (*P*<0.001), STAT1(*P*<0.05), S100A8 (*P*<0.0001), S100A9(*P*<0.0001) and CCND1(*P*<0.0001) were significantly lower while those of USP-18 (*P*<0.0001), PARP9 (*P*<0.01) and ANXA1(*P*<0.0001) were significantly higher in BT-474 cells. The mRNA levels of RBL2, SASH1 and PGR were not significantly different between MCF-7 and BT-474 cells. Compared with those of MCF-7 cells, the mRNA levels of EIF2AK2 (*P*<0.001), TGM2 (*P*<0.0001), DDX58 (*P*<0.0001), RBL2 (*P*<0.0001), SASH1 (*P*<0.0001), PARP9 (*P*<0.05), STAT2 (*P*<0.0001), STAT1 (*P*<0.05), PGR (*P*<0.0001), S100A8 (*P*<0.0001), S100A9(*P*<0.0001) CCND1 (*P*<0.0001) and ANXA1 (*P*<0.0001) were all significantly lower in ZR-75-1 cells. The mRNA level of USP-18 (*P*<0.001) was significantly higher in ZR-75-1 cells than in MCF-7 cells.

The mRNA expression profiles of these genes in two ER^-^-breast cancer cell lines were quite different. As compared to those of MCF-7, the mRNA levels of EIF2AK2 (*P*<0.0001), USP-18 (P<0.001), RBL2(P<0.01), SASH1(P<0.0001), PARP9 (P<0.001), S100A8 (*P*<0.0001), S100A9 (*P*<0.0001) and ANXA1 (P<0.001) were significantly higher while those of TGM2 (*P*<0.0001), DDX58 (*P*<0.0001), STAT2 (*P*<0.01), PGR (*P*<0.0001) and CCND1 (*P*<0.0001) were significantly lower in MDA-MB-468 cells. No significant difference in STAT1 mRNA level was detected between MCF-7 and MDA-MB-468 cells. Compared with those of MCF7, the mRNA levels of EIF2AK2 (*P*<0.05), TGM2 (*P*<0.0001), DDX58 (*P*<0.001) and ANXA1 (p<0.001) were significantly higher while those of USP-18 (*P*<0.0001), SASH1 (*P*<0.001), PGR (*P*<0.0001) S100A8 (*P*<0.0001), S100A9 (*P*<0.0001) and CCND1 (*P*<0.0001) were significantly lower in MDA-MB-231 cells. The mRNA levels of RBL2, PARP9, STAT2 and STAT1 were not significantly different between MCF-7 and MDA-MB-231. It is interesting to note that the mRNA levels of EIF2AK2 and ANXA1 were significantly higher while those of PGR and CCND1 were significantly lower in two ER^-^- breast cancer cell lines than in MCF-7 cells.

### The mRNA expression profiles of differentially expressed genes in ER^+^- and ER^-^-breast tumor tissues and their corresponding tumor-adjacent tissues

**Figure 5:**
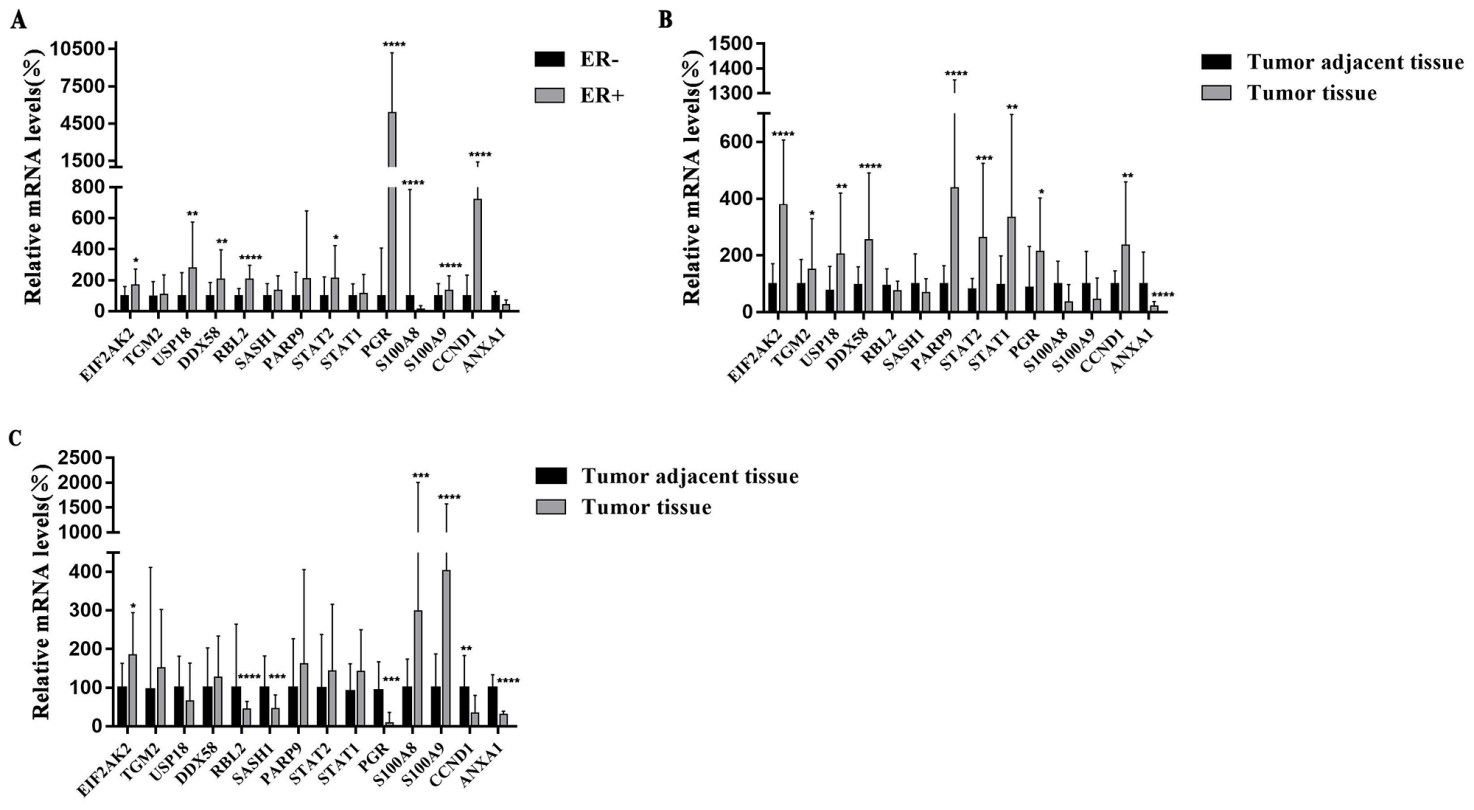
**(A)** Relative mRNA levels of 14 genes in ER^+^ breast cancer tissues and ER^-^ breast cancer tissues. The samples were obtained from 55 patients with ER^+^ breast cancer (n=27) and ER^-^ breast cancer (n=28). **(B)** Relative mRNA levels of genes in ER^+^ cancer tissues (n=27) and their corresponding tumor adjacent tissues (2 cm from the tumor site). **(C)** Relative mRNA levels of 14 genes in ER^-^ cancer tissues (n=28) and their corresponding tumor-adjacent tissues (2 cm from the tumor site). All data are presented as median with interquartile range. ^*^P <0.05, ^**^P <0.01, ^***^P <0.001 ^****^P <0.0001 vs. controls.

We also compared the relative mRNA levels of 14 genes between ER^+^ - and ER^-^-breast cancer tissues obtained from breast cancer patients. The results shown in Figure 5A indicated that mRNA levels of EIF2AK2 (*P*<0.05), USP18 (*P*<0.001), DDX58 (*P*<0.001), RBL2 (*P*<0.0001), STAT2 (*P*<0.05), PGR (*P*<0.0001), S1000A9 (*P*<0.0001) and CCND1 (*P*<0.0001) were significantly higher in ER^+^- than in ER^-^-breast cancer tissues while the expression level of S100A8 (*P*<0.0001) was significantly lower in ER^+^- than in ER^-^-breast cancer tissues. The mRNA levels of TGM2, SASH1, PARP9, STAT1 and ANXA1 were not significantly different between ER^+^ and ER^-^-breast cancer tissues. These results are quite different from those obtained from comparison of ER^+^ MCF-7 with those of two ER^-^-cell lines. These differences are likely due to the heterogeneity and diversity of the ER^+^ and ER^-^-breast cancer tissues from patients with different backgrounds.

We compared the mRNA expression profiles of these 14 genes between ER^+^ breast cancer tissues versus their corresponding tumor-adjacent tissues derived from 27 patients with ER^+^-breast cancer (Figure 5B). It can be seen from Figure 5B that the mRNA levels of EIF2AK2 (*P*<0.0001), TGM2 (*P*<0.05), USP18 (*P*<0.01), DDX58 (*P*<0.0001) PARP9 (*P*<0.0001), STAT2 (*P*<0.001), STAT1 (*P*<0.001), PGR (*P*<0.05) and CCND1 (*P*<0.01) were all significantly higher in cancer tissues than in their corresponding tumor-adjacent tissues. It is worth noting that all of these genes except PGR (*P*<0.05) and CCND1 (*P*<0.01) which were down-regulated, were up-regulated in ER^+^ MCF-7 cells by 4-OH-TAM, confirming their pathophysiological relevance and possible involvement in mediating the anti-breast cancer effects of TAM. The mRNA level of ANXA1 (*P*<0.0001) was extremely and significantly lower in tumor tissues than in the corresponding tumor-adjacent tissues. It is worth pointing out that as shown above, the mRNA level of ANXA1 was significantly down-regulated in 4-OH-TAM-treated MCF-7 cells. The mRNA levels of RBL2 (*P*=0.2687), SASH1 (*P*=0.0859), S100A8 (*P*=0.2198) and S100A9 (*P*=0.0731) were not significantly different between ER^+^-cancer tissues and ER^+^-tumor-adjacent tissues.

We also compared the mRNA profiles of these 14 genes between ER^-^ -breast tumor tissues and their corresponding tumor-adjacent tissues derived from 28 patients with ER^-^-breast cancers. The results shown in Figure 5C indicated that the mRNA levels of RBL2 (*P*<0.0001), SASH1 (*P*<0.001), PGR (*P*<0.001), CCND1 (*P*<0.01) and ANXA1 (*P*<0.0001) were all significantly lower in ER^-^-cancer tissues than in their corresponding tumor-adjacent tissues while those of EIF2AK2 (*P*<0.05), S100A8 (*P*<0.001) and S100A9 (*P*<0.0001) were significantly higher in ER^-^-cancer tissues than in their corresponding tumor-adjacent tissues. The mRNA levels of TGM2, USP18, DDX58, PARP9, STAT1 and STAT2 were not significantly different between ER^-^-tumor tissues and their corresponding tumor-adjacent tissues.

When the mRNA levels of 14 genes between ER^+^-tumor tissues and their corresponding tumor adjacent tissues and between ER^-^-tumor tissues and their corresponding tumor-adjacent tissues were compared, several interesting and important points can be pointed out as follows: (a) while the mRNA levels of USP18 and DDX58 were significantly higher in ER^+^-cancer tissues than in their corresponding tumor-adjacent tissues, they were not significantly different between ER^-^-tumor tissues and their corresponding tumor adjacent tissues; (b) while the mRNA levels of RBL2 and SASH1 were not significantly different between ER^+^ tumor tissues and their corresponding tumor adjacent tissues, they are extremely and significantly lower in ER^-^-tumor tissues than in their corresponding tumor adjacent tissues; (c) The mRNA levels of S100A8 and S100A9 were lower and there were no significant difference between ER^+^-tumor tissues and their corresponding tumor-adjacent tissues. However, the mRNA levels of S100A8 and S100A9 are significantly higher in ER^-^-cancer tissues than in their corresponding tumor-adjacent tissues; and (d) only EIF2AK2 mRNA levels were significantly higher in both ER^+^- and ER^-^-tumor tissues than in their corresponding tumor-adjacent tissues whereas only the mRNA levels of ANXA1were significantly lower in both ER^+^- and ER^-^-breast tumor tissues than their corresponding tumor-adjacent tissues.

### Bio-informatics analysis: Classic pathways

**Figure 6:**
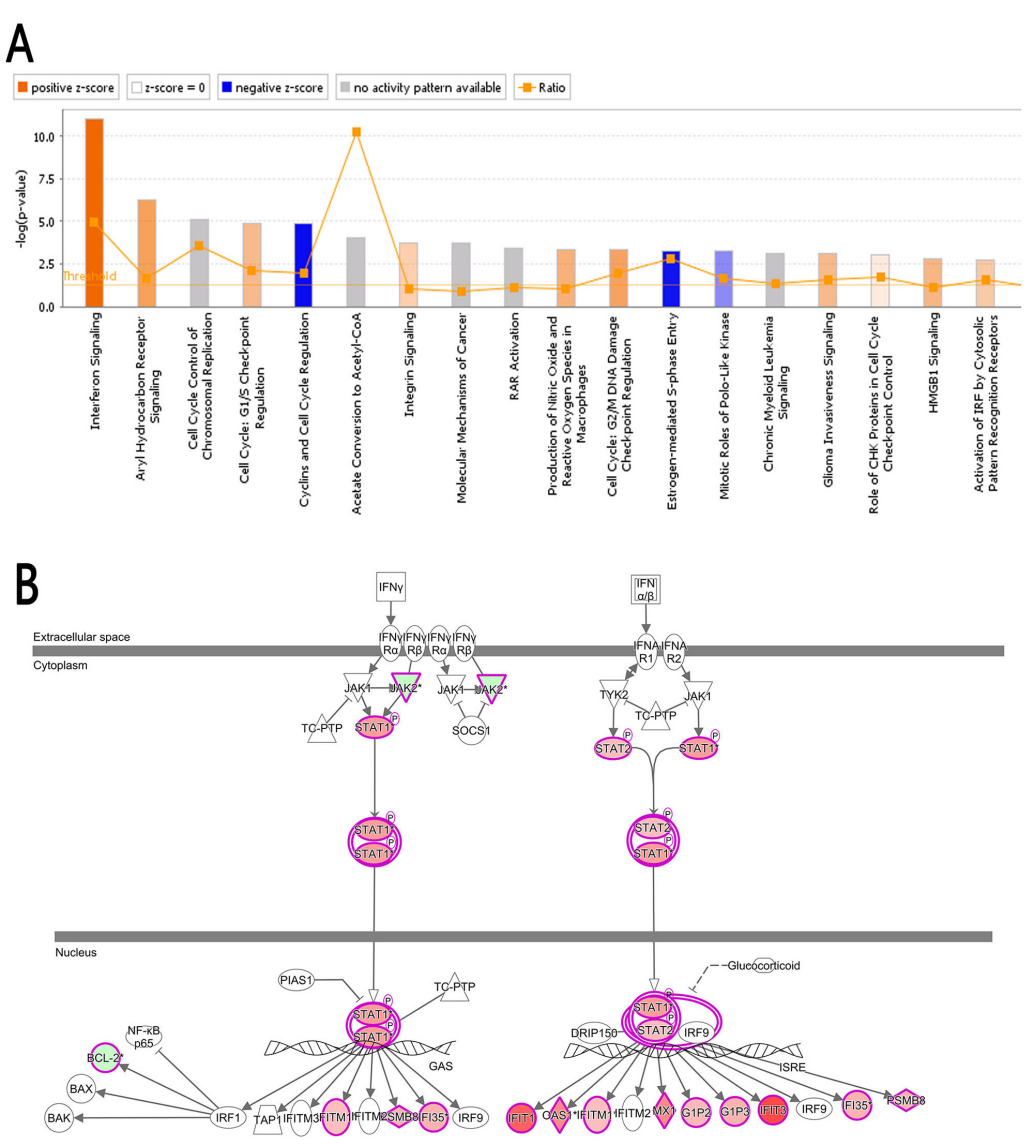
Classical pathway **(A)** The cluster status of the differentially expressed genes in classical signal transduction pathways. The classical signal transduction pathways regulated by the differentially expressed genes were summed up by 800 signal transduction and metabolism pathways gathered and summarized via IPA. All the signal pathways were ranked by using –log (P-value). **(B)** The interferon signal pathway; the canonical interferon signal pathway showed the signaling process of the associated molecule and differentially expressed genes. The highlighted molecules represent the differentially expressed genes, red in varying degrees corresponding to the different degree of up-regulation, green in varying degrees corresponding to the different degree of down-regulation.

Ingenuity^®^ Pathway Analysis (IPA^®^), a confluence analysis software (www.ingenuity.com) [37, 38], is an analysis tool that uncovers the significance of ‘Omics’ data and identifies new targets or candidate biomarkers within the context of biological systems. Figure 6A shows the Classical Pathways, which demonstrates the cluster status of the differentially expressed genes in classical signal transduction pathways. The signal pathways shown in orange color indicate Z-score>0; the signal pathways shown in blue color indicate Z-score<0; Z-score>2 indicated that the pathway is significantly activated. Z-score<-2 indicated that the pathway is significantly inhibited. Ratio represents the ratio of the number of the differentially expressed genes to the number of all the genes in that signal pathway. In this study, the interferon signal pathway (Figure 6B) was significantly activated as the Z-score of this pathway was 3.051. The analysis of upstream regulatory factors indicated the upstream regulatory factors of all the differentially expressed genes identified in this study. Base on the concatenation of 41 and 96 genes, IFNA2 was predicted to be strongly activated whereas β-estradiol was predicted to be strongly inhibited (Table 4).

**Table 4:** The upstream regulatory factors predicted by the differentially expressed genes

Genes (fold change)	Upstream regulator (predicted activation state)
TXNIP (2.693), TUBG1 (-1.537), TSC22D3 (1.706), TPM1 (3.48), TNS3 (2.193), TIMP3 (-1.771), THBS1 (-2.63), THBD (1.513), TGFB2 (1.996), TFF1 (-8.121), STON1 (1.837), SMC2 (-1.556), SMAD3 (1.579), SLC39A6 (-1.791), SLC25A15 (-1.641), SLC22A5 (-1.523), SKP2 (-1.581), SIAH2 (-2.051), SERPINA3 (-2.487), S100A7 (-3.398), RND3 (1.866), RFC4 (-1.523), RERG (-2.806), RBL2 (1.922), RAMP3 (-2.416), RAD54L (-1.518), RAB31 (-1.86), PTTG1 (-1.559), PRSS23 (-6.334), POLE2 (-1.54), PLK2 (1.691), PKIB (-2.626), PIK3R3 (1.505), PGR (-4.753), PDZK1 (-1.893), OXTR (-1.755), ODC1 (-1.766), NUDT1 (-1.639), NRP1 (2.291), NR4A1 (-1.573), NPY1R (-4.334), NDRG1 (-2.091), MYO1B (2.154), MYBL1 (-2.614), MYB (-2.368), MXD4 (1.554), MCM7 (-1.513), MB (1.6), LHFPL2 (1.997), LDLR (-1.551), KITLG (-1.665), KCTD6 (-1.911), ITGAE (-1.527), INHBB (1.923), IL1R1 (3.252), IGFBP4 (-1.922), IGFBP3 (1.567), IGF2 (-1.678), HTRA1 (-1.536), HMGCS1 (-1.524), GREB1 (-13.924), GNS (1.533), GK (-1.578), GAL (-4.313), GAB2 (-2.236), FOXC1 (-2.738), FOS (-2.902), FADS1 (-1.745), EGR3 (-3.418), EFNA1 (1.542), DUSP10 (2.302), DNMT3B (-1.715), CYP24A1 (-1.564), CYP1A1 (2.667), CXCL12 (-3.468), CLSTN2 (-1.941), CDC25A (-1.535), CDC20 (-1.619), CCND1 (-1.83), CCNB2 (-1.527), CAV1 (-1.911), C8orf44-SGK3/SGK3 (-8.98), C3 (-1.635), BTG2 (2.085), BTG1 (1.671), BMP4 (1.505), BIK (1.867), BCL2 (-1.993), BCAS1 (3.124), ASCL1 (-7.006), ARL4A (1.862), AREG (-6.828), APOD (1.994), ANXA3 (1.906), ANXA1 (-2.307), ABCC5 (1.84)	β-estradiol (Inhibited)
XAF1 (4.874), USP18 (1.861), UGT1A6 (2.738), UBE2L6 (2.187), TRIM14 (1.668), TNFSF10 (5.984), TGM2 (1.923), STAT1 (2.36), SP110 (2.416), SP100 (3.8), SDC1 (1.621), SAMHD1 (1.632), SAMD9 (2.661), PPP2R2C (1.532), PLSCR1 (2.069), PARP9 (2.657), PARP12 (1.55), OAS3 (2.187), OAS2 (5.481), OAS1 (2.793), NT5E (3.149), MX1 (2.775), LGALS3BP (1.901), ISG15 (1.669), IFITM1 (1.524), IFIT5 (1.736), IFIT3 (4.302), IFIT2 (2.246), IFIT1 (3.769), IFI6 (1.817), IFI44L (4.28), IFI44 (2.135), IFI35 (1.839), IFI27 (1.519), HERC6 (1.569), EIF2AK2 (1.765), DDX60 (3.177), DDX58 (2.337), CMPK2 (3.124), CDKN2B (2.153), C19orf66 (1.839)	IFNA2 (Activated)

### Bio-informatics analysis: the network of β-estradiol

**Figure 7:**
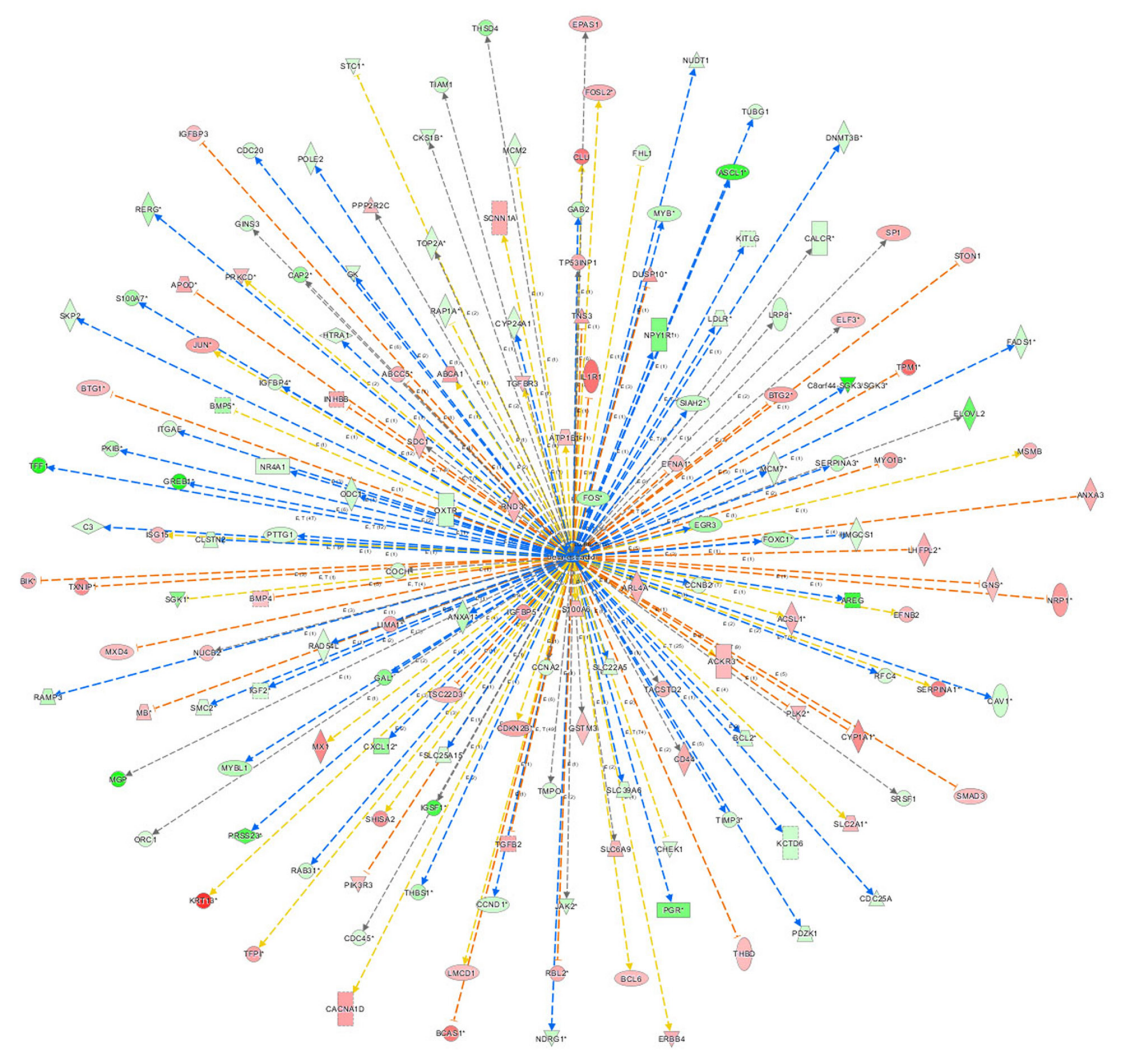
The network of β-estradiol The network of β-estradiol showed the network of predicted upstream regulatory factor, β-estradiol and down-stream molecules in the data set. The orange lines indicate the activating expression status between the upstream regulatory factors and the downstream genes; blue lines indicate the inhibitory expression status between the upstream regulatory factors and down-stream genes; the grey lines indicate that there is no prediction information related to the expression status in the data.

Figure 7 showed the network of upstream regulatory factor of β-estradiol, which illustrated the interrelationships between the upstream regulatory factors and the down-stream molecules co-existing in the data set. The orange lines indicate the activating expression status between the upstream regulatory factors and the downstream genes; blue lines indicated the inhibitory expression status between the upstream regulatory factors and down-stream genes; the grey lines indicated that there was no prediction information related to the expression status in the data set. For instance, β-estradiol can enhance BCL-6 mRNA level. But in the experimental data, BCL-6 level was significantly down-regulated. The expression status between β-estradiol and PGR was concomitant.

### Bio-informatics analysis: disease and function analysis

**Figure 8:**
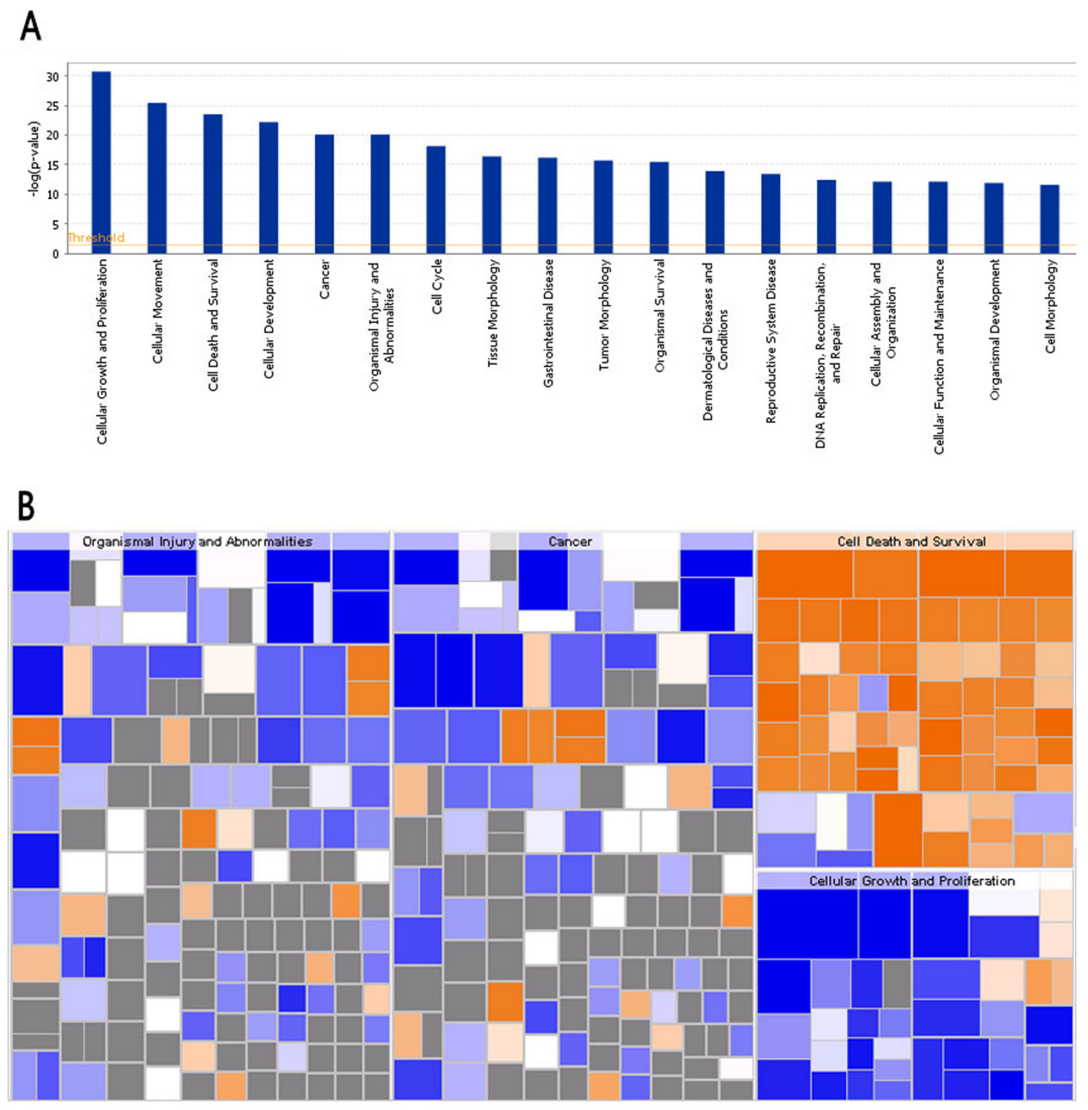
Bio-informatics analysis of the disease and function **(A)** The disease and function bar figure illustrate the cluster status of the differential genes in categories of disease and functions. All the diseases and functions were ranked by using –Log (P-value). **(B)** Disease and Function Heat Map illustrates the relationships between up-regulation and down regulation of the differentially expressed genes and the activation and inhibition of functions and diseases. The orange color indicates Z-score>0, blue color indicates Z-score<0, grey color indicates Z-score value. Z-score>2 indicates that function is strongly activated; Z-score<-2 indicates that that function is strongly inhibited. In this study, the functions that are strongly activated include: apoptosis of cervical cancer cell lines (3.597 folds), cell death of cervical cancer cell lines (3.268 folds) and the functions that were significantly inhibited include: proliferation of cancer cells (-3.510 folds), and proliferation of cells (-3.239 folds).

Disease and Function Bar Figure (Figure 8A) illustrates the cluster status of the differentially expressed genes in the categories of diseases and functions. Disease and function Heat Map (Figure 8B) illustrates the relationships between up-regulation and down-regulation of differentially expressed genes and the activation and inhibition of functions and diseases. As shown in Figure 8B, the functions that are strongly activated by 4-OH-TAM treatment included apoptosis of cervical cancer cell lines (3.597 folds) and cell death of cervical cancer cell lines (3.268 folds) whereas the functions that were significantly inhibited included proliferation of cancer cells (-3.510 folds) and proliferation of cells (-3.239 folds).

### Bio-informatics analysis: gene function networks

**Figure 9:**
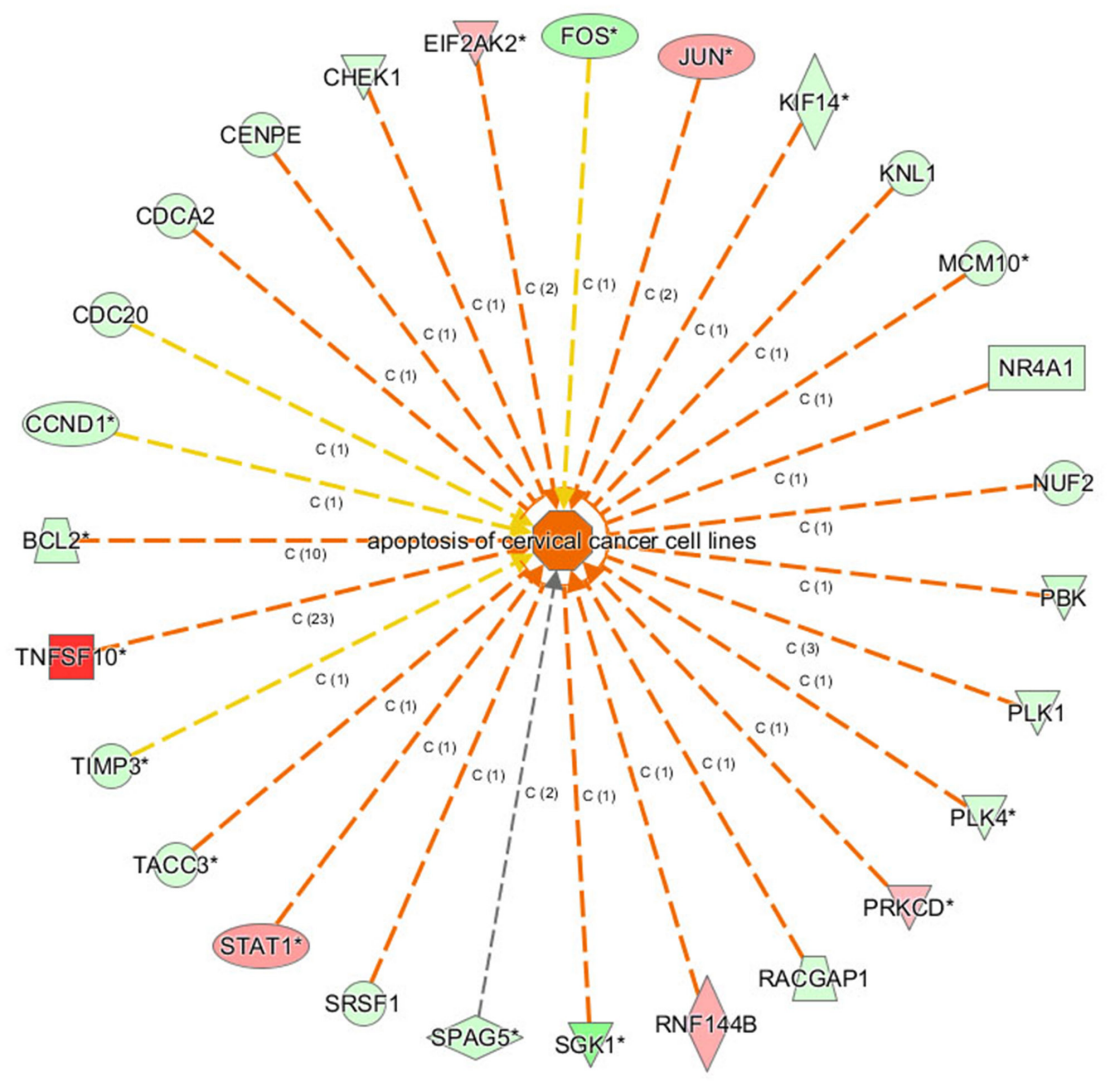
The gene function network The orange lines indicate the concomitantly activated expression status between the upstream regulatory factors and the genes; blue line indicates the concomitantly inhibited expression status between the upstream regulatory factors and the genes; Yellow lines indicate that the expression state between the upstream regulatory factors and genes is inconsistent. The grey lines indicated that there no exists the prediction information about the expression state.

The gene function networks shown in Figure 9 illustrate the interrelationships between activation and inhibition of genes and functions. The networks illustrate all the genes with concentrated data and the assigned function or disease and give their up- or down-regulation relations and based on literature in Ingenuity knowledge pool to support the predicted interaction relationship. Figure 9 showed that apoptosis of cervical cancer cell lines was significantly activated. Its |Z-score| was ranked the first among the categories of function and diseases.

### Bio-informatics analysis: gene regulation effect network analysis

**Figure 10:**
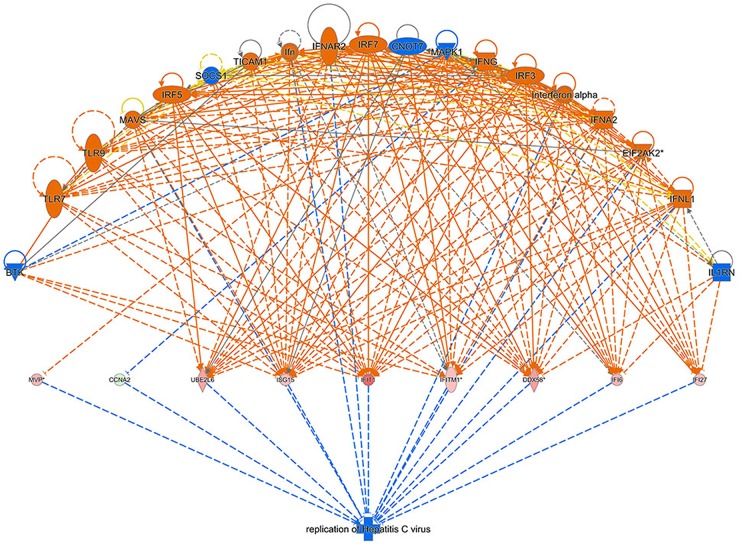
Regulator effect network analysis The data set and disease and the Consistency Score is calculated for each regulator effect network, where higher scores are awarded to networks that are directionally consistent, meaning that most of the paths from regulator to target to disease/function are consistent with the predicted state of the regulator, the observed direction of expression. The higher the score is, the more accurate the result of the Regulator Effect is.

Regulator Effect Network Analysis shown in Figure 10 illustrates the possible action pathways, including the upstream regulatory network and downstream function, in which the differentially expressed genes participate. The consistence and connection of the causal relationships among the up-stream regulatory factors, differentially expressed genes and diseases and functions in the network were measured by Consistency Score. The higher the score is, the more accurate the result of the regulator effect is. In our result, the significantly and differentially expressed genes (CCNA2, DDX58, IFI27, IFI6, IFIT1, IFITM1, ISG15, MVP, and UBE2L6 etc.) caused by 4-OH-TAM treatment predicted the possible upstream regulators, including BTK, CNOT7, EIF2AK2, IFN, IFNA2, IFNAR2, IFNG, IFNL1, IL1RN, Interferon-α, IRF3, IRF5, IRF7, MAPK1, MAVS, SOCS1, TICAM1, TLR7, and TLR9 with the highest Consistency Score, which predicted the inhibition of Hepatitis C virus.

### Bio-informatics analysis: gene interaction networks

**Figure 11:**
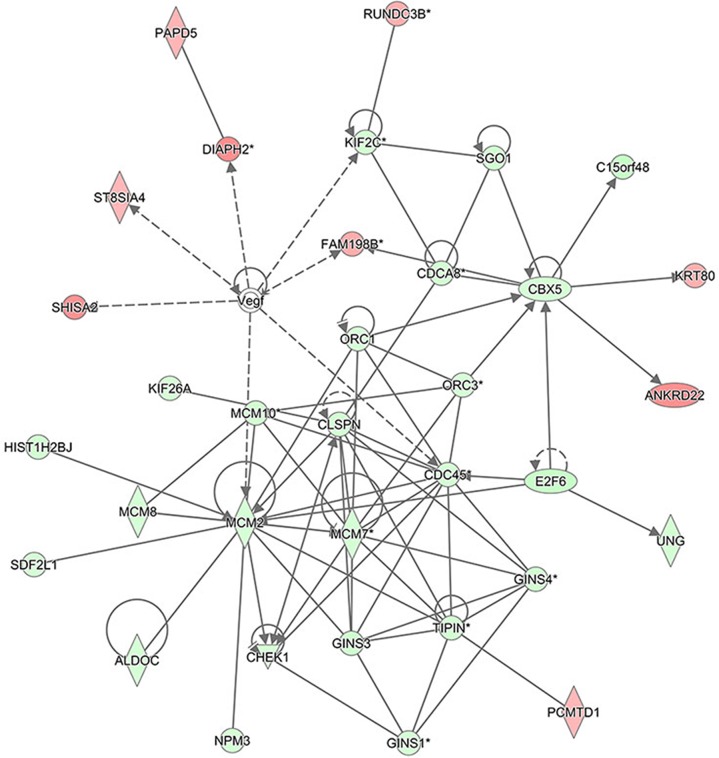
The interaction network analysis The interaction network illustrates the interrelationships among the molecules with concentrated data. This network mainly affects DNA replication, recombination, and repair, cell cycle, and embryonic development. In this network, the molecules in red represent the up-regulated expression while in green represent the down-regulated expression. The solid lines represent the direct interactions, the dotted lines represent the indirect interactions, the solid lines with arrow represent the direct activation.

The interaction network analyses shown in Figure 11 illustrate the interrelationships among the molecules with concentrated data. IPA uses network generation algorithm to divide the inter-molecule network into multiple networks and gives score for each network. The scoring is based on hypergeometric distribution. The negative log of the significant level was obtained through r-Fisher's exact test. All the networks were ranked based on their scores. The networks that were ranked first were those that affect DNA replication, recombination, and repair, cell cycle, and embryonic development. Gene Interaction Network illustrates the interrelationship networks among the molecules in the defined functional domain. In this network, genes, proteins and chemical substances were expressed with different shapes. This network was ranked the number 1 network in this study.

## DISCUSSION

TAM has been clinically used to treat patients with ERα-positive breast cancer. However, the molecular mechanisms underlying its anti-breast cancer effects have not been fully elucidated. The present study was conducted to examine the inhibitory effects of 4-OH-TAM on growth and proliferation of breast cancer cells, to identify novel 4-OH-TAM-induced gene signatures in both ER^+^- and ER^-^-breast cancer cell lines as well as in ER^+^- and ER^-^-breast tumor tissues and their corresponding tumor-adjacent tissues, aiming to elucidate the mechanisms underlying the anti-cancer effects of TAM. This study led to several interesting and novel findings.

First, we observed that MCF-7 cell growth was suppressed by 4-OH-TAM treatment in a dose-dependent manner with IC_50_ of 29 μM. Consistent with this result, IC_50_ values of 4-OH-TAM in MCF-7 and MDA-MB 231 cells were reported to be 27 μM and 18 μM, respectively [39]. IC_50_ values of TAM for MCF-7 parents and MCF-7 sub-lines with different degrees of TAM resistance ranged from 0.39 to >10 μM [40]. 4-OH-TAM at 1×10^-7^ M caused nearly 10% inhibitions on MCF-7 cell growth, which is in agreement with previously reported observation that 4-OH-TAM at 10^-7^ M significantly repressed E2-induced proliferation of MCF-7 cells [41]. With increasing 4-OH-TAM concentrations, the growth rate was further reduced and the inhibitory effect reached maximal, which was 94.4% reduction, when the concentration was increased to 3.33 × 10^-4^ M. The growth inhibition of MCF-7 cells is mainly due to the inhibition on cell proliferation and induction of apoptosis and necrosis induced by 4-OH-TAM. The anti-proliferation effects of TAM are mainly related to its blockage of ERα-mediated growth stimulatory effects of β-estradiol (E2), a pivotal regulator of cell growth proliferation in the normal breast and breast cancer cells. This is supported by the Classic Pathways Analysis results indicating that E2 is predicted to be strongly inhibited (Table 4).

One of the molecular mechanisms underlying the anti-cancer effects of 4-OH-TAM can be related to the actions of 4-OH-TAM on the functional networks of E2- and c-Myc-responsive genes, including those gene signatures with known or predicted roles in cell cycle control, cell growth, cell death/survival signaling and transcriptional regulation [42]. We observed that 4-OH-TAM up-regulated the anti-proliferation genes, including B cell translocation gene 1 (BTG1) and BTG2. BTG1 expression was down-regulated in breast cancer cells and significantly correlated with proliferation, poor overall survival, histological grade, clinic stage, and lymph node metastasis by regulating protein expression levels of Cyclin-D1, Bcl-2, and MMP-9 [43]. BTG2 possesses anti-proliferation and anti-invasion functions in human lung cancer cells [44]. Both BTG1 and BTG2 may act as the negative regulator to breast cancer cells. Furthermore, we also observed that PGR gene, which encodes PR, was down-regulated by 4.753 folds while CCND1, which encodes cyclin D1, was down-regulated by 1.709 folds in 4-OH-TAM-treated MCF-7 cells, which was confirmed by qRT-PCR analysis (Figure 3). PR has been used as a biomarker of ERα function and breast cancer prognosis. PR bound to ERα to direct ERα chromatin binding events at cyclin D1/MYC promoters within breast cancer cells, leading to a unique gene expression program, which was associated with good clinical outcome in the presence of the agonist ligands and blockage of ERα with the pure anti-estrogen fulvestrant disrupted the interaction between ERα and PR *in vitro* and suppressed MPA-dependent tumor growth *in vivo* [45]. Blockage of ERα-pathways with TAM caused similar effects, leading to the decreased expression levels of cyclin D1 and C-myc. Progesterone inhibited E2-mediated growth of ERα-positive xenografts and primary ERα-positive breast tumor explants, and increased anti-proliferative effects when being coupled with an ERα antagonist. PGR loss is commonly seen in ERα-positive breast cancers, explaining lower PR levels in a subset of patients. Together, these findings indicate that PR functions as a molecular rheostat to control ERα chromatin binding and transcriptional activities, such as Cyclin D1 and c-Myc implicated in prognosis and therapeutic interventions [46]. Furthermore, our results showed that mRNA levels of both CCND1 (Figure 4, panel M) and PGR (Figure 4, panel 4J) were significantly lower in ER^-^-MDA-MB-468 and MDA-MB-231 cells than those of MCF-7 cells. We also observed: (a) the mRNA levels of both CCND1 and PGR were significantly higher in ER^+^-breast tumor tissues than in ER^-^-breast tumor tissues (Figure 5A); (b) the mRNA levels of both CCND1 and PGR were significantly higher in ER^+^-breast tumor tissues than in their corresponding tumor-adjacent tissues (Figure 5B); and (c) they were significantly lower in ER^-^-breast tumor tissues than in their corresponding tumor-adjacent tissues (Figure 5C). Altogether, these results further reveal that both CCND1 and PGR are involved in mediating the anti-breast cancer effects of TAM in ER^+^-breast cancer tissue.

Another important mechanism involved in the anti-proliferation effects of 4-OH-TAM may be attributed to TAM-inducted activation of interferon signal transduction pathway [47]. Signal transducer and activator of transcription 1 (STAT1) and STAT2 are required for the anti-proliferative effects of both interferon-α (INF-α) and INF-γ[48]. We made following interesting, important and novel observations: (a) the mRNA levels of both STAT1 and STAT2 in MCF-7 cells were significantly up-regulated by 4-OH-TAM treatment; (b) the mRNA levels of STAT2 was significantly higher in ER^+^-MCF-7 cells than in ER^-^- MDA-MB-468 cells; (c) the mRNA levels of STAT2 but not STAT1 were significantly higher in ER^+^- than ER^-^-breast tumor tissues (Figure 5A); (d) mRNA levels of STAT2 and STAT1 were significantly higher in ER^+^-breast tumor tissues than in their corresponding tumor-adjacent tissues (Figure 5B) but they were not significantly different between ER^-^-tumor tissues and in their corresponding tumor-adjacent tissues (Figure 5C). These results indicate that 4-OH-TAM-inducted activation of STAT2 and STAT1 in interferon signal transduction pathway may contribute to anti-breast cancer effects of TAM.

A combination of TAM and interferon was initially used to treat advanced breast cancer [49]. In TAM-treated MCF-7 cells, IFN-β and IFN-γ more readily activated INF-stimulated gene factor-3 (ISGF-3) [50]. Moreover, we observed that in addition to STAT1 and STAT2, the genes encoding IFI27, IFI35, IFI6, IFIT1, IFIT2, IFIT3 and IFITM1 were also concomitantly up-regulated by 4-OH-TAM treatment. Consistent with this observation, it was reported [51] that TAM up-regulated the expression levels of immune response-related genes, including INF-inducible genes (IFITM, IFIT1, IFNA1, MXI and GIP3) in cultured normal human mammary epithelial cells.

Type I IFNs (IFN-α/β) have been used for treatment of some types of cancer, hepatitis B/C, and multiple sclerosis. They regulate the expression of proteins with anti-proliferative, pro-apoptotic, and pro-inflammatory and anti-viral functions through activation of receptor-associated JAK1 and TYK2 [52]. STAT1 and STAT2 are involved in IFN-α and IFN-γ signaling and cytokine-mediated biological responses [53]. They are recruited to the IFN-α/β receptor and become tyrosine phosphorylated by JAKs. After being activated, they bind to each other as either STAT1 homodimers or heterodimers to form the ISGF3 complexes with IRF9, which translocate to the nucleus and initiate transcription of ISGs. STAT1 homodimer binds to a γ-activated region [54] whereas ISGF3 complex binds to an IFN-stimulated response element (ISRE) region in the promoter of ISGs [55]. STAT1-STAT2 heterodimers and ISGF3 have the cooperative DNA-binding activities, which contribute to the transcriptional activation of IFN-α-responsive genes [56]. Both STAT1 and STAT2 are involved in IFN-induced apoptosis [57–61] via their SH2 domain [61]. IRF-1 serves as transcription activator of genes induced by INF-α, INF-β and INF-γ and is critical for TAM-mediated apoptosis in human mammary epithelial cells. The anti-tumor actions of IFN have great potential implications for cancer therapy [58]. Together, these findings indicate that 4-OH-TAM significantly activates INF signal pathways and that up-regulation of ISGs can be one of the important aspects in the anti-tumor activity of this drug combination.

We observed that a number of apoptosis-related genes were significantly up-regulated in MCF-7 cells by 4-OH-TAM. As shown in (Table 4), in the disease and functional annotations within the categories of Cell Death and Survival, various apoptosis, cell death and necrosis, and differentiation were activated with Z-scores in the range from 2.025 to 3.597. A number of apoptosis-related genes, including BCL2, BCL6, BIK, CD-44, CDC-20, CDCA2, CDC25 and CDC45, are associated with the changes in these functions. It was reported that among 12 TAM-regulated genes identified, testis enhanced gene transcript Bax inhibitor-1 (TEGT-BI-1) was down-regulated in tumor tissues of TAM-treated patients [33]. The expression levels of both TEGTBI-1 and CD63 were down-regulated in tumor tissues of patients treated with TAM. TEGTB1 inhibits the expression of Bax, which promotes apoptosis. On the other hand, CD63 encodes a cell membrane protein involved in platelet activation, cell adhesion and cell motility. TAM may modulate tumor growth by down-regulating genes involved in cell cycle control, tumor invasion and metastasis. CD-44 is involved in apoptotic response and promotion of disease development in chronic lymphocytic leukemia [62]. CCD-20 can suppress apoptosis through targeting Bim for ubiquitination and destruction [63]. CDC25 A is involved in the regulation of cell proliferation and inhibition of apoptosis [64]. It was also reported that TAM induced apoptosis of MCF-7 cells by inducing the expression and secretion of transforming growth factor beta 1 (TGF-β) through ER [65, 66] and that TAM induced c-Myc expression in ER-negative MDA-231 cells. These observations suggest that the effects of TAM on ER-negative breast cancer cells may be mediated through c-Myc overexpression and that C-Myc may play a critical role in the growth and progression of MDA-231 breast cancer cells [42]. Thus, the functional networks between E2- and c-Myc-responsive genes are related to TAM therapy in breast cancer.

We observed that the expression of ubiquitin-like specific protease 18 (USP18) was up-regulated by 1.861 and 3.806 folds by 4-OH-TAM. USP-18 mRNA levels were significantly higher in BT-474, ZR-75-1 and MDA-MB468 cells than in MCF-7 cells but was lower in MDA-MB-231 cells (Figure 4, panel 4C). USP-18 mRNA level was significantly higher in ER^+^- than in ER^-^-breast tumor tissues (Figure 5A) and in their corresponding tumor-adjacent tissues (Figure 5B). But no significant difference in USP-18 mRNA level was detected between ER^-^-tumor tissues and their corresponding tumor-adjacent tissues (Figure 5C). USP18, a member of the ubiquitin-specific proteases family of enzymes cleaving ubiquitin from ubiquitinated protein substrates, is an INF-stimulated gene 15-specific protease functioning as a negative regulator of IFN α/β signaling pathway and is specifically induced by viral infection and IFNα/β[67]. Mice lacking this gene were hypersensitive to INF, suggesting that it down-regulates INF signal pathways and plays an important role in the host innate immune response and inflammation [68]. Burkart et al. [69] reported that USP18 deficient mammary epithelial cells created an anti-tumor environment driven by hypersensitivity to IFN-λ and elevated secretion of CXCL-10, a member of the chemokine family induced in a variety of cells in response to INF-γ and lipopolysaccharide. CxcL-10 possessed strong angiostatic activity [70] and acted as a chemoattractant for Th1 subtype T cells [71]. In Usp18 null mice, the growth of mammary tumor, angiogenesis and invasiveness of mammary epithelial tumor cells were reduced. Tumors of USP18 deficient mice also displayed an increased CD^4+^ T-cell infiltration, an increased level of cxcL-10 and hypersensitivity to IFN-λ enhanced up-regulation of CxcL-10 expression, which created a Th1/M1-polarized cytokine tumor environment and inhibited tumor progression [69]. It appears that treatment of MCF-7 cells with 4-OH-TAM induces IFN α/β signaling pathway and indirectly induces USP18, its negative regulator. The ultimate outcome of its anti-breast cancer effects may be dependent on the balance between IFN α/β signaling pathway and USP18-mediated cascade. The increased expression of USP18 induced by 4-OH-TAM may serve as the negative regulator for preventing the hypersensitivity to IFN-λ, enhanced up-regulation of CXCL-10 and other pathways induced by IFN α, β and γ.

It is worth noting that USP18 is also involved in antiviral activity of INF. For instance, silencing USP18 expression with siRNA potentiated the antiviral activity of INF against hepatitis C virus (HCV) infection [72]. ISG15 is an ubiquitin-like protein modifier, which conjugates to target proteins (ISGylation) via the sequential enzymatic action of activating E1, conjugating E2, and ligating E3 enzymes. ISGylation modulates signal transduction pathways and host antiviral response. ISGylation process is reversible through the action of USP18, an ISG15 protease. ISG15/USP18 pathway plays important roles in response to chronic HCV infection. HCV may exploit the ISG15/USP18 pathway to promote viral replication and evade innate anti-viral immune responses through suppressing IFN signaling pathway [73]. HCV represents a prevalent and major health concern in most parts of the world. Males and females experience different responses to HCV infection and show variations in response to IFN-based therapy. This gender difference may be attributed to sex hormones. TAM has an antiviral effect against HCV. E2 was reported to be able to inhibit the expression of IFN-stimulated gene MxA in HCV-infected peripheral blood mononuclear cells (PBMCs) whereas pretreatment of PBMCs with TAM reversed the suppressive effect of E2 on the JAK-STAT pathway in IFNα-treated PBMCs. TAM-pretreatment also significantly up-regulated MxA expression in imiquimod-treated PBMCs, independent of ER blocking and an up-regulation in TLR7 expression [74]. Thus, up-regulation of USP18 by 4-OH-TAM treatment may inhibit, at least in part, INF signal pathways in to respond to chronic HCV infection. In this regards, this aspect needs to be taken into consideration when TAM is used to treat patients with ER^+^-breast cancer, though TAM's anti-HCV activity was found to abrogate the functional association of ER with viral RNA polymerase NS5B [75].

In this study, we found that S100A8 and S100A9 genes were down-regulated by 4.562 and 3.560 folds in MCF-7 cells treated with 4-OH-TAM. Compared to those in MCF-7 cells, the mRNA levels of S100A8 and S100A9 were significantly lower in ER^+^ BT-474 and ZR-75-1 cells and in ER^-^- MDA-MB-231 cells but were significantly higher in ER^-^- MDA-MB-468 cells (Figure 4, panels 4K and 4L). The mRNA levels of S100A8 were significantly lower but that of S100A9 was significantly higher in ER^+^-breast tumor than in ER^-^-breast tumor (Figure 5A). While no significant difference in mRNA levels of S100A8 and S100A9 were detected between ER^+^-breast tumor tissues and their corresponding tumor-adjacent tissues (Figure 5B); The S100A8 and S100A9 were highly expressed and their mRNA levels were significantly higher in ER^-^-breast tumor tissues than in their corresponding tumor-adjacent tissues (Figure 5C). Both S100A8 and S100A9 are members of the S100 family of calcium-binding proteins. They contain 2EF hand calcium-binding motifs and exert various calcium-mediated cell growth, differentiation, migration and signal transduction. They are overexpressed in many human tumors, such as carcinomas of glandular cell origin and their over expression is associated with poor pathological parameters in invasive ductal carcinoma of the breast [76, 77]. High level of S100A9 but not that of S100A8 was found to be associated with loss of ER and the poor overall survival of breast cancer patients and to be involved in the poor prognosis of Her^2+^/basal-like subtypes of breast cancer [78]. S100A8 and S100A9 are inflammatory chemoattractants and their expression levels can be induced by distant primary tumors, attract macrophage antigen 1 (Mac 1)-positive myeloid cells in the pre-metastatic lung. Tumor cells utilize this mechanism to acquire migration activity with pseudopodia for invasion through activation of the mitogen-activated protein kinase (MAPK) p38. Their expression levels in lung Mac-1 positive-myeloid cells and endothelial cells were up-regulated by endothelial growth factor A, TNF-α and TGF-β both *in vitro* and *in vivo* [79]. Overexpression of S100A9 in cultured embryonic stem cells or transgenic mice led to inhibition of differentiation of dendritic cells and macrophages and induced accumulation of myeloid-derived suppressor cells, one of the major immunological abnormalities in cancer. Neutralizing anti-S100A8 and anti-S100A9 antibodies blocked the morphological changes and migration of tumor cells and Mac 1-positive myeloid cells [79]. Similarly, knocking down their expression significantly inhibited the invasive and migratory phenotypes of human gastric cancer SNU484 cells by inhibiting MMP-2 expression [80]. Thus, down-regulation of S100A8 and S100A9 genes by 4-OH-TAM can be related to the inhibition of the invasive and migratory phenotypes of human breast cancer cells, particularly in ER^-^-breast cancer cells.

In this study, we found that the expression of ANXA1 (annexin A1) was significantly down regulated by 4-OH-TAM treatment. Compared to that of MCF-7 cells, ANXA1 mRNA levels were significantly higher in MDA-MB-468 and MDA-MB-231 cells (Figure 4, panel 4N). While those of ANXA1 were not significantly different between ER^+^- and ER^-^-breast tumor tissue (Figure 5A), they were significantly lower in both ER^+^- and ER^-^-breast tumor tissues than in their corresponding tumor-adjacent tissues (Figure 5B and 5C). Annexin-1 is calcium and phospholipid binding protein, acting as a strong inhibitor of glucocorticoid-induced eicosanoid synthesis and phospholipase A2 (PLA2), and is involved in regulating cell death signaling, phagocytic clearance of apoptotic cells. Annexin A1 plays a role in regulation of growth arrest induced by high levels of estrogen in MCF-7 breast cancer cells via acting as a tumor suppressor involved in modulation of the proliferative functions of estrogens [81, 82] while decreased its expression was associated with breast cancer development and progression [83]. Trastuzumab is an effective therapeutic agent for patients with HER2-positive breast cancer. Breast tumors with low levels of the ANXA1 displayed a benefit from trastuzumab. However, high levels of ANXA1 were related to a resistance to trastuzmab. Thus, ANXA1 may be predictive of trastuzumab resistance in patients with HER2-positive breast cancer [84]. Down-regulation of ANXA1 by 4-OH-TAM treatment points to the possibility that co-treatment of breast cancer cells with trastuzmab and TAM can enhance the sensitivity of breast cancer cells to trastuzmab.

In summary, the experimental, and qRT-PCR verification results and bioinformatics analysis demonstrated that 4-OH-TAM potently inhibited MCF-7 cell growth, up-regulated genes involved in INF signaling, cyclines, and cell cycle regulation, apoptosis, indicating that these genes may be involved in diseases and functions, including cell proliferation, apoptosis and proliferation of cancer cells. 4-OH-TAM also down-regulated a number of genes, including PGR, S100A8, S100A9, CCND1 and ANEXA1, which are involved in IFN α/β signaling pathway and immune/inflammatory response to viral infection. This study also revealed that the expression profiles of 4-OH-TAM-induced genes identified in MCF-7 cells displayed some similar but also quite different expression profiles among three ER^+^- and two ER^-^-breast cancer cell lines and between ER^+^- and ER^-^-breast tumor tissues and their corresponding tumor-adjacent tissues obtained from Chinese breast cancer patients. This study has paved a good foundation for subsequent studies to further elucidate the cellular and molecular mechanisms underlying the anti-breast cancer effects of TAM in both E2/ER-dependent and independent pathways, particularly in Chinese breast cancer patients.

## MATERIALS AND METHODS

### Major reagents used in this study

CCK-8 (Cat # 96992) was purchased from Sigma (St. Louis, MS, USA). Fetal bovine serum (FBS) was purchased from Ausbian (Sydney, Australia). DMEM (10-013–CVR) was purchased from Corning Inc. (Corning, NY, USA). Trypsin (Cat # T4665) was purchased from Sinopharm Chemical Reagent Co., Ltd. (Shanghai, China). D-Hanks Hanks was obtained from Shanghai GeneChem Co. Ltd. (Shanghai, China). 4-hydroytamoxifen (4-OH-TAM) was purchased from Sigma (St. Louis, MS, USA). TRIZOL RNA Isolation Kit (Catalog #: 12183555) was purchased from Thermo Fisher Scientific (Waltham, MA, USA). M-MLV (M1705), dNTPs (U1240) and RNase inhibitor (N2115) were obtained from Promega (Madison, WI, USA). Oligo dT (B0205) was obtained from Sangon Biotech Co., Ltd. (Shanghai, China). Bulge-LoopTM miRNA qPCR Primer Sets were synthesized by Ibibio (Guangzhou, Guangdong, China). Reverse and forward primers were synthesized by Shanghai Genechem Co., LTD (Shanghai, China). SYBR Master Mixture (DRR041B) was obtained from TAKARA (Daliang, Liaoning, China). Reagents for reverse transcription were purchased from Axygen (Union City, CA, USA).

### Equipment and devices

Nanodrop 2000 UV-Vis spectrophotometer was purchased from Thermo Scientific. The Agilent 2100 Bioanalyzer was obtained from Agilent technologies (Santa Clara, CA, USA). Electrophoretic apparatus (EPS-600) was purchased from Tannon (Shanghai, China). Ultrafine homogenizer (F6/10) was purchased from FLUKO Equipment Shanghai Co., Ltd. (Shanghai, China); Real time PCR LightCycler480 was purchased from Roche (Basel, Switzerland).

### Cell culture and treatments with 4-OH-TAM

MCF-7 cells were obtained from American Type Culture Collection (ATCC) (Manassas, VA, USA) and maintained and cultured in Dulbecco's modified Eagle's medium (DMEM) (Cellgro, Manassas, VA, USA) supplemented with 10% FCS and 1% penicillin/streptomycin (Cellgro) at 37°C incubator in 5% CO_2_ atmosphere. In addition, two ER^+^ (BT-474 and ZR-75-1) and two ER^-^ (MDA-MB-468 and MDA-MB-231) cells obtained from the Cell Bank of the Chinese Academy of Sciences (Shanghai, China) were also used in this study. BT-474 and ZR-75-1 were cultured in Gibco^™^ RPMI Media 1640 while MDA-MB-468 and MDA-MB-231 cells were cultured in Leibovitz's L-15 Medium. All of the complete mediums were supplemented with 10% FBS and 1% penicillin/streptomycin. BT-474 and ZR-75-1 cells were cultured at 37°C incubator in 5% CO_2_ atmosphere. MDA-MB-468 and MDA-MB-231 were cultured at 37°C incubator in a humidified atmosphere of 100% air.

### Human breast tumor tissues and their tumor-adjacent tissues

#### Patients and tissue samples

A total of 55 patients with primary breast cancer (all females aged between 42 and 83 years, median age of 58 years) who were hospitalized in the Third Affiliated Hospital of Soochow University from September 2015 to May 2016 were recruited in the present study. The protocols of the present study were approved by the Institutional Ethics Committee of the Third Affiliated Hospital of Soochow University [(2014)KENo.121] and all the patients gave their written informed consent to use of their specimens and data. All the patients underwent modified radical operations. All the tumor tissue samples and their corresponding tumor-adjacent tissue samples (2 cm from the tumor site) were excised and quickly frozen in liquid nitrogen after resection and preserved at −80°C until use for subsequent pathophysiological analyses.

#### Determination of ERα status via immunohistochemistry

The human breast tumor and their tumor-adjacent tissues were fixed with formalin and embedded in paraffin. Formalin-fixed and paraffin-embedded tissues were cut into 3-μm-thick consecutive sections, de-waxed in xylene and rehydrated in graded ethanol solutions. Corresponding polyclonal mouse antibodies against human ERα and PR (MXB Biotechnologies, Fuzhou, China) were used. There were two steps in EnVision IHC staining and color development, 3, 3’-diaminobenzidine (DAB) was used as the color reagent, and phosphate-buffered saline was used as substitute for the primary antibodies and taken as the negative control. Tumors were considered positive when there were at least 1% of positively stained tumor nuclei in the sample on testing in the presence of expected reactivity of internal (normal epithelial elements) and external controls [85, 86]. Immunostaining images were scored independently by two pathologists. The staining intensity (I) was graded on a scale of 0–3^+^ with 0 representing no detectable staining and 3^+^ representing the strongest staining. The four strongest staining regions were randomly selected under a 400× field. In each of the four regions, the rate of positive cell staining (R) under a 400 x field was calculated and defined as follows: 0. no staining; 1, ≤10% tumor cells with staining; 2, 11-50% tumor cells with staining; 3, 51-75% tumor cells with staining; and 4, >75% tumor cells with staining. Samples with scores <3 were considered negative, while those with scores ≥3 were considered positive. Histochemistry score= I × R [87]. All the tissue samples (27 cases of ER^+^-breast cancer and 28 cases of ER^-^ -breast cancer) were histopathologically examined. They were all defined as invasive ductal breast cancer. In the present study, no patient was given any treatments before surgery.

#### Treatment of MCF-7 cells with 4-OH-TAM

For treatment of MCF-7 cells with 4-OH-TAM, the experiments were carried out in phenol red-free DMEM supplemented with 10% charcoal-treated FBS, 50 μg/mL streptomycin, 50 UI/ml penicillin and 2 mM l-glutamine (Gibco Invitrogen). When MCF-7 cells grew to exponential phase, they were detached by treatment with 0.25% trypsin for 5 min. The detached cell suspension was centrifuged at 200x g and 4°C for 8-10 min. The cell pellets were re-suspended with complete culture medium. The cell numbers were counted with cell counting chamber. In this study, two cell densities, i.e. 4000 cells/well and 8000 cells/well, were used with 5 wells for each treatment group at each time point. Cell suspension (200 μL) containing 4000 or 8000 cells were seeded in each well. After having settled, the cells were visualized under microscope to check the evenness. If the cell densities in different groups were not evenly distributed, one group was fixed and the other groups were micro-tuned. The cells were re-seeded until they were evenly distributed. If the cell numbers in control groups were too high, then the cell number was reduced and the cell culture plate was reseeded. The cells were cultured in Cell Culture Incubator at 37°C with supply of 5% CO_2_. MCF-7 cell suspension containing 4000 cells in 200 μL/well and 8000 cells in 200 μL/well corresponding to 20% and 40% confluences per well of the 96-well plate were seeded. In the next day, the cells in different groups were treated with 4-0H ATM dissolved in dimethyl sulfoxide (DMSO) at 0, 1×10^-7^, 3.33×10^-7^, 1×10^-6^, 3.33×10^-6^, 1×10^-5^, 3.33×10^-5^, 1×10^-4^, 3.33×10^-4^, 1×10^-3^ and 3.33×10^-3^ (Table 1), respectively, and incubated for 72h. Thereafter, the cell viability in each group was determined by CCK-8 assay with commercial CCK-8 kit, a sensitive colorimetric assay that can be used for the determination of cell viability in both cell proliferation and cytotoxicity assays. WST-8, 5-(3-carboxymethoxyphenyl)-2-(4,5-dimethylthiazolyl)-3-(4-sulfo phenyl) tetrazolium, inner salt (CCK-8), a highly water-soluble tetrazolium salt [34], is a substrate of dehydrogenase. Within the cells, WST-8 is reduced by the mitochondrially localized dehydrogenase to give a highly water soluble, yellow-color formazan dye, whose amount is directly proportional to the number of living cells. Formazan dye can be measured with Micro-plate ELISA Analyzer at 450 nm and OD value can indirectly reflect the number of viable cells. When being compared to that of the control group, the cell-killing ability of 4-OH-TAM can be reflected. Briefly, at 2h prior to termination of the experiment, 10μL of CCK-8 was added to each well without changing the culture medium. At 4 h later, the cell culture plate was placed on the shaker and shaken for 2-5 min and the absorbance density (OD) at 450 nM (OD_450_) was measured with a micro-plate reader. The cell numbers of each treatment group were calculated according to the OD_450_ value.

#### Extraction, quantitation and quality verification of total RNA

MCF-cells were treated without or with 4-OH-TAM at 1.0 × 10^-7^ M for 72 h. The 4-OH-TAM at 1×10^-7^ M was used to treat MCF-7 cells for microarray analysis. This dose was selected on our preliminary results as well as the previous observation that 4-OH-TAM at this dose significantly suppressed estrogen-induced proliferation of MCF-7 cells [41]. After being treated with 4-OH-TAM at 1×10^-7^ M, total RNA of MCF-7 cells was extracted according to the Isolation Protocols provided in TRIZOL RNA Isolation Kit as follows. The cells were harvested and centrifuged at 2000 rpm for 5 min. The supernatant was removed. 1 mL of Trizol was added. After being fully mixed, the solution was settled at room temperature for 5 min and then transferred to a fresh 1.5 mL Eppendorf (EP) tube. After 200 μL of chloroform was added to each tube, the tube was shaken up and down for 15 seconds and settled down at room temperature for 10 min. The mixed solution was centrifuged at 12800 rpm and 4°C for 15 min. The supernatant was carefully withdrawn and transferred to a fresh 1.5 mL EP tube. The equal volume of pre-cold isopropanol was added. After being mixed evenly, the mixed solution was settled down at 4°C in ice for 10 min and then centrifuged at 12800 rpm and 4°C for 12 min. The supernatant was discarded. The RNA pellet was washed with 1 mL of 75% ethanol (freshly prepared with DECP-treated water) and centrifuged at 12800 rpm and 4°C for 5 min. A large majority of supernatant was discarded and tube was then centrifuged again at 12800 rpm and 4°C for 5 min. The supernatant was completely discarded and RNA sample was air-dried at room temperature for 5 min. When the RNA sample became semi-transparent, RNase-free water was added to dissolve the air dried RNA samples (the volume of water added depended on the amount of RNA). After being completely dissolved, the concentration and quality of the isolated total RNA from MCF-7 cells of TAM-treated groups and control (NC) group were quantified with NanoDrop 2000 (Thermo Scientific, Waltham, MA, USA). The quality and integrity of total RNA were checked with Agilent Bioanalyzer 2100. The values of A260/A280 ratio of all the total RNA samples were in the range of 1.97-2.05, the Rin values were in the range of 8.1-9.6 and the values of the 28S/18S ratio were in the range of 1.5-1.8, indicating that total RNA samples isolated from MCF-7 cells of both groups were well qualified for being used in gene expression analysis with Gene Expression Array.

Total RNA was isolated from 30 mg of ER^+^-human breast tumor samples (n=27) and ER^-^-human breast tumor samples (n=28) and their corresponding tumor adjacent tissue samples (2 cm from the tumor site) using RNA Isolation Kit (Cat # R6734) purchased from OMEGA (GA, USA) according to the instructions provided by the manufacture. Determination of the integrity, quality and concentration of RNA samples isolated from human breast tumor tissues and their corresponding tumor-adjacent tissues were performed the same as described above.

#### Analysis of gene expression profiles induced by 4-OH-TAM treatment with human gene expression array

GeneChip^®^ PrimeView^™^ Human Gene Expression Array (part # 901838) was used in this study. This array provides a comprehensive coverage of the human genome in a cartridge array format designed for use with the GeneChip^®^ Scanner 3000 7G series.

The raw microarray data of PrimeView^™^ Human Gene Expression Array, which provides a comprehensive coverage of the human genome in a cartridge array format designed for use with the GeneChip^®^ Scanner 3000 7G series, was applied to Genespring GX predictor algorithm (Santa Clara, CA, USA) to be analyzed. The analysis excluded the probe sets which signal intensities was less than 20% and the probe sets which variable coefficient was larger than 25%. The qualified data was normalized by RMA algorithm and then log-transformed followed by median-subtraction. The gene expression data were processed with unpair *t*-test to identify the differentially expressed genes and the false discovery rate (FDA) was calculated through Benjamini-Hochberg method. The genes were regarded as differentially expressed when their FDRs were less than 0.05 and the fold change was larger than 1.5.

### Microarray hybridization, scanning, and data acquisition

#### Complementary DNA (cDNA) synthesis

cDNA synthesis was performed in the reaction system consisted of 5 μL of 5× RT buffer, 2 μL of 10 mM dNTPs (dATP, dGTP, dCTP and dTTP), 0.4 μL of Rnasin (40 U/μL), 1 μL of M-MLV-RTase (200 U/μL) and 5.6 μL of RNase-free H_2_O. This reaction system was incubated at 42°C water bath to allow reaction for 1 h, and transferred at 70°C water bath and incubated for 10 min to inactive reverse transcriptase. The cDNA products obtained were stored at -20°C freezer for subsequent use and analysis.

#### Reverse transcription of mRNA into cDNA was performed as follows

1 μL Oligo dT (0.5 μg/μL) and 2.0 μg total RNA were added into small PCR tube and brought up to 10 mL with RNase-free H_2_O. After mixed well, the tube was centrifuged briefly and incubated at 70°C water both for 10 min and then quickly in an ice bath (mixture of water and ice) to allow annealing of Oligo dT and template. To this mixture, 4 μL of 5× RT buffer, 2 μL of 10 mM dNTPs, 0.4 μL of Rnasin (40 U/μL, 1 μL of M-MLV-RTase (200 U/μL) and 2.6 μL of RNase-free H2O. This reaction system was incubated at 42°C water both to allow reaction for 1 h, and then transferred to 70°C water both and incubated for 10 min to inactivate reverse transcriptase. The obtained cDNA was stored at -20°C for subsequent use and analysis.

The cDNA (500 ng) samples prepared with mRNA from MCF7 cells treated with or without 4-OH-TAM were labeled by random priming with incorporation of Cyanine 5-dUTP for the tester DNA and Cyanine3-dUTP for the driver samples, respectively. GeneChip 3′IVT Expression Kit was chosen to perform reverse transcription, double-stranded DNA template conversion, and *in vitro* transcription for a cRNA synthesis and labeling. Fluorescent probes were hybridized, washed, and stained with GeneChip Hybridization Wash and Stain Kit. Then, the samples were mixed, concentrated by evaporation under vacuum and re-suspended in pre-hybridization buffer, with Denhardt's solution replacing BSA. The two-labeled cDNA mixtures were hybridized with the arrayed slides overnight at 42°C. The slides were then washed for 5 min with 1 SSC-1% SDS, 3 min with 1 SSC, 3 min with 0.1 X SSC for 1 min with water, and finally with 95% ethanol-dried. Accurate differential measurements (final fluorescence ratios) were expressed as the average of nine independent assays where each sequence was arrayed in triplicate. Visualization, quantification and gene expression analysis were performed with GENEPIX 3.0 software (AXON)(Union City, CA, USA). Significantly and differentially expressed genes between MCF-7 cells treated with 4-OH-TAM and control cells were defined as genes with absolute log transformed fold change (abs(logFC))>1.5, at the significance level P<0.05. The data were normalized by the autonormalization method described previously [38].

#### Validation of the quality of microarray data

GeneChip^®^ PrimeView^™^ Human Gene Expression Array was applied to investigate the gene expression profiles of MCF-7 cells induced by 4-OH-TAMtreatment at 1×10^-7^ M for 72 h. In order to ensure the quality and the reliability of the microarray data, we firstly validated the quality of the microarray data obtained by conducting analysis on (a) Signal Histogram, (b) Relative box, (c) Pearson's correlation of the signals and (d) Principal component analysis (PCA). These data were provided as the Supplementary Materials. Supplementary Figure 1A showed the Signal Histogram, which demonstrated the statistical distribution of the expression levels of all the chip probes. Each curve represents the statistics of the number of probes in different expression value intervals. The better the overlap ratio of the signal distribution curve is, the more reliable the microarray experiment is. Signal Histogram indicates that all the chip results are highly reliable. Supplementary Figure 1B showed the Relative Signal Box Plot, which demonstrated the distribution of normalized log-transformed expression signal. Supplementary Figure 1C represents the Pearson's Correlation coefficient diagram of all the 8 samples according to the expression signal, which indicated the inter-chip correlation level between each two chips. Supplementary Figure 1D represents the Principal Component Analysis (PCA), which displayed the three-dimensional distribution of all the samples according to three main variables. Each spot in the figure represents one sample, which demonstrated the intra-group similarity and the inter-group difference. There are high intra-NC group and intra-TAM group similarities and there was large difference between the 4-OH-TAM-treated group and NC group.

#### Verification of Up-regulated and down-regulated genes by quantitative real-time PCR

Isolation of total RNA from five breast cancer cell lines (MCF-7, BT-474, ZR-75-1, MDA-MB-468 and MDA-MB-231) and cDNA synthesis via reverse transcription were performed the same as those descried for microarray assay. Total RNA from human breast cancer tissues and corresponding adjacent tissues was isolated with DNA/RNA/Protein Isolation Kit (Cat # R6734) purchased from OMEGA (GA, USA). cDNA synthesis with total RNA from human breast tumor tissues and tumor adjacent-tissues via reverse transcription were performed the same as those descried for microarray assay.

The mRNA levels of the 14 target genes and reference genes were measured under real-time PCR using TaqMan technology. The PCR primer sets for target genes and reference genes and corresponding probes were designed according to the gene sequence information of GenBank and listed in Table 5. GAPDH was used as reference gene. The real-time PCR reaction for each gene was performed in a 25 μL volume, containing 0.1 μL of 100 μM each primer and probe, 2 μL of cDNA, 2.5 μL of 10×buffer, 2.5 μL of MgCl_2_ (25 mM), 0.25 μL of dNTP (10 mmol/L), and 0.5 μL of Taq DNA polymerase. Thermal cycling conditions included the following steps: initial denaturation at 95°C for 3 min, followed by 40 cycles at 95°C for 5 sec and 60°C for 15 sec. All the PCR assays were performed on the LightCycler (Roche, Switzerland) real-time PCR system. Quantification of target genes mRNA levels was performed by normalizing to GAPDH mRNA level by F=2-ΔΔCt.

**Table 5:** Sequences of primers and probes for quantitative real-time PCR

Gene	Primer/probe	Sequence (5’ to 3’)
STAT1	Forward primer	GACCGAGCAGAGGCGACC
	Reverse primer	CACAGAGTGCGAACGTTAACCTAG
	Probe	FAM-AGCGCGCTCGGGAGAGGCT-BHQ1
STAT2	Forward primer	ATACTAGGGACGGGAAGTCGC
	Reverse primer	CGCCATTTGGGCTCTGATT
	Probe	FAM-ACCAGAGCCATTGGAGGGCGC-BHQ1
EIF2AK2	Forward primer	CTGAAAAATGATGGAAAGCGAAC`
	Reverse primer	GAATTAGCCCCAAAGCGTAGAG
	Probe	FAM-CTTTGCGATACATGAGCCCAGAACAG-BHQ1
TGM2	Forward primer	CACCCACACCTACAAATACCCAG
	Reverse primer	CCCTGTCTCCTCCTTCTCGG
	Probe	FAM-TCCTCAGAGGAGAGGGAGGCCTTCA-BHQ1
DDX58	Forward primer	CGGAAGACCCTGGACCCTAC
	Reverse primer	AAAAAGTGTGGCAGCCTCCAT
	Probe	FAM-ACATCCTGAGCTACATGGCCCCCT-BHQ1
PARP9	Forward primer	GAAATGTCCTGTGCCTCCAACT
	Reverse primer	ACCTCATTGTCTATCTTCTCCACCTT
	Probe	FAM-AACCTGCAAACCACATTTTTCAAACTGT-BHQ1
SASH1	Forward primer	TGAGCGATGAGGAGCGGAT
	Reverse primer	CCAGTCAGCAGGGTCCAGG
	Probe	FAM-CGACTGCCGGTGCTGGGCCTC-BHQ1
RBL2	Forward primer	TGCTGCCTTGAGGTCGTCAC
	Reverse primer	GCCATCTTCTGCTCTAATGAATACTT
	Probe	FAM-TTCTTATAAGCCTCCTGGGAATTTTCCA-BHQ1
USP18	Forward primer	TGCCCAACTGTACCTCAAACTCT
	Reverse primer	CCTTCACCCGGATCGTATACAG
	Probe	FAM-CAGATCACTGATGTGCACTTGGTGGA-BHQ1
CCND1	Forward primer	TCCATGCGGAAGATCGTCG
	Reverse primer	CGGCTCTTTTTCACGGGCT
	Probe	FAM-ACCTGGATGCTGGAGGTCTGCGA-BHQ1
S100A9	Forward primer	TCTGTGTGGCTCCTCGGCT
	Reverse primer	TGATGGTCTCTATGTTGCGTTCC
	Probe	FAM-TGACAGAGTGCAAGACGATGACTTGC-BHQ1
S100A8	Forward primer	GCTAGAGACCGAGTGTCCTCAGTAT
	Reverse primer	ACTGCACCATCAGTGTTGATATCC
	Probe	FAM-AAGGGTGCAGACGTCTGGTTCAAAGA-BHQ1
ANXA1	Forward primer	GCCAAAGACATAACCTCAGACACAT
	Reverse primer	CACACCAAAGTCCTCAGATCGG
	Probe	FAM-TGGAGATTTTCGGAACGCTTTGCTT-BHQ1
PGR	Forward primer	TGTCATTATGGTGTCCTTACCTGTG
	Reverse primer	TGCGGATTTTATCAACGATGC
	Probe	FAM-AGAGGGCAATGGAAGGGCAGCAC-BHQ1

### Statistical analysis

Parametric data are shown as means ± standard deviation (SD) or means ± SEM, whereas nonparametric data are reported as median with interquartile range. Parametric data were analyzed by analysis of variance and subsequently by unpaired *t*-test. Nonparametric data were analyzed by Wilcoxon matched-pairs signed-rank test or Mann–Whitney tests. Statistical analyses were performed with the GraphPad Prism 6.0 software (GraphPad Software, Inc., San Diego, California, USA). The difference between groups with *P* < 0.05 was considered significant.

## SUPPLEMENTARY MATERIALS FIGURES


