# Supplementary material for: Identification of differentially expressed genes in human breast cancer cells induced by 4-hydroxyltamoxifen and elucidation of their pathophysiological relevance and mechanisms
Source: Oncotarget. 2017 Dec 20;9(2):2475–501. doi: 10.18632/oncotarget.23504 (PMC5788654; doi:10.18632/oncotarget.23504)
Supplement: Supplementary file 1 [file oncotarget-09-2475-s001.pdf]

Identification of differentially expressed genes in human breast cancer cells induced by 4-hydroxyltamoxifen and elucidation of their pathophysiological relevance and mechanisms

SUPPLEMENTARY MATERIALS

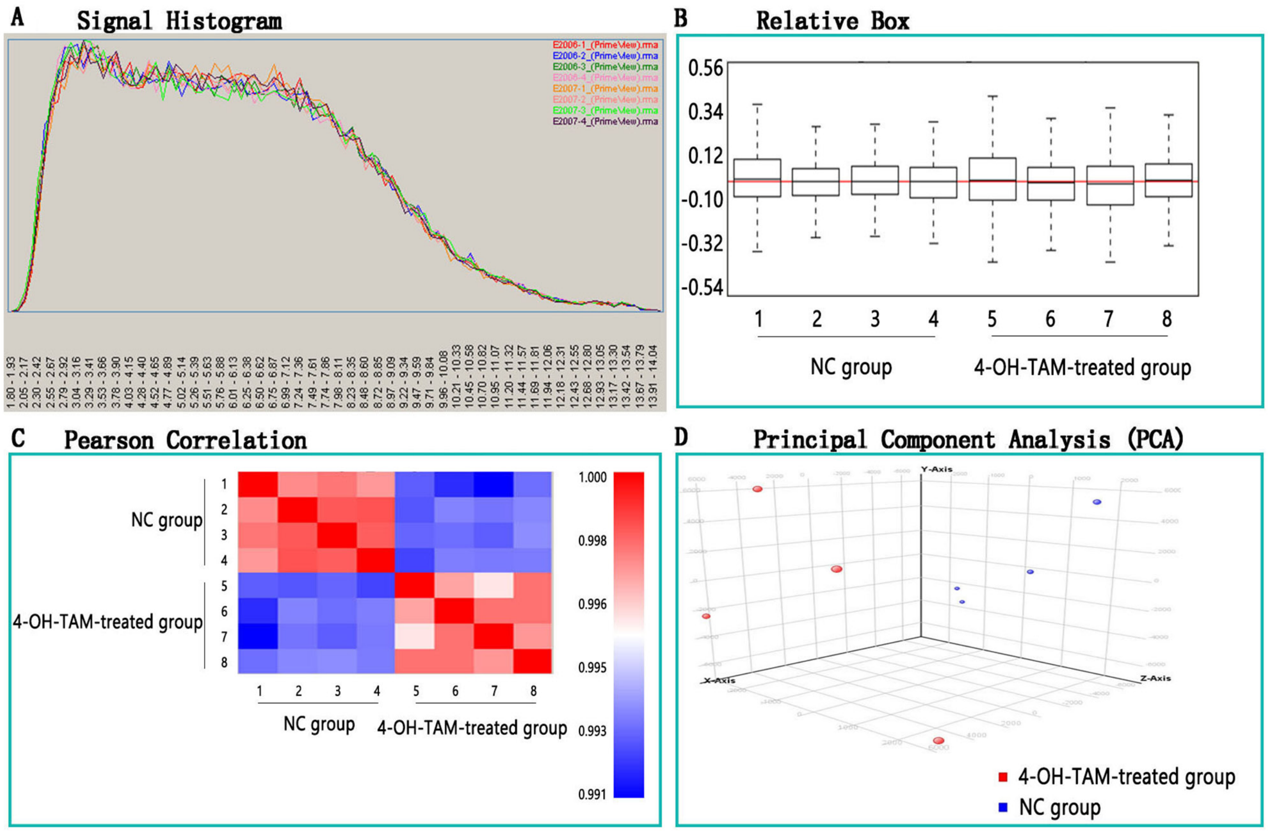

Supplementary Figure 1: (A) Signal histogram, which demonstrates the statistical distribution of the expression levels of all the chip probes. The X-axis represents the expression value interval while the Y-axis represents the probe statistic within the expression value interval. The legends were shown in the bar chart in left upper corner. Each curve represents the statistics of the number of probes in different expression value intervals. The better the overlap ratio of the signal distribution curve is, the more reliable the microarray experiment is. The Signal Histogram indicates that all the chip results are highly reliable. **(B)** The relative box, which demonstrates the distribution of relative logarithm expression value of all the chips. The X-axis represents the names of sample and the Y-axis represents the relative logarithm expression value. The middle red line represents the mean value of the relative logarithm expression of all the samples. The upper cross lines and the lower cross lines in each box represents the upper and lower 90% confidence intervals, respectively. The upper and lower edges of the box represent the upper and the lower quartiles. The middle black line indicates the median position. The closer the distribution of the relative logarithm expression value is, the higher the repeatability of the close data is. (B) showed that repeatability of all the samples in this study is very high. **(C)** Pearson's correlation of the signals, which indicate the inter-chip correlation level of all the chips. Each square box indicates the correlation level between X-axis coordinate and the Y-axis coordinate corresponding to two samples. This figure shows the Pearson's correlation. When the correlation coefficient is 1.0, the correlation is completely positive. The closer the correlation coefficient to 1.0 is, the higher the correlation is. In this study, there are high correlations among samples in the NC group. There are also high correlations among samples in 4-OH-TAM-treated group. Their correlation coefficients were all higher than 0.95. **(D)** The principal component analysis (PCA), which exhibited the distributions of three main variables of all the samples. Each spot in the figure represents one sample. The legends were shown in the right lower Conner. (D) demonstrated the intra-group similarity and the inter-group difference. The higher the cluster degree among the samples is, the higher the similarity is and wise versus. There are high similarity of intra- NC group and intra-TAM group. There was large difference between the TAM-treated group and NC group.
